# Multi‐Spectroscopic Interrogation of the Spatial Linker Distribution in Defect‐Engineered Metal–Organic Framework Crystals: The [Cu_3_(btc)_2−*x*_(cydc)_*x*_] Showcase†

**DOI:** 10.1002/chem.201905645

**Published:** 2020-03-03

**Authors:** Miguel Rivera‐Torrente, Matthias Filez, Florian Meirer, Bert M. Weckhuysen

**Affiliations:** ^1^ Inorganic Chemistry and Catalysis Debye Institute for Nanomaterials Science Utrecht University Universiteitsweg 99 3584 CG Utrecht The Netherlands

**Keywords:** copper, crystal engineering, materials characterization, metal–organic frameworks, microporous materials, Raman spectroscopy

## Abstract

In the past few years, defect‐engineered metal–organic frameworks (DEMOFs) have been studied due to the plethora of textural, catalytic, or magnetic properties that can be enhanced by carefully introducing defect sites into the crystal lattices of MOFs. In this work, the spatial distribution of two different non‐defective and defective linkers, namely 1,3,5‐benzenetricarboxylate (BTC) and 5‐cyano‐1,3‐benzenedicarboxylate (CYDC), respectively, has been studied in different DEMOF crystals of the HKUST‐1 topology. Raman micro‐spectroscopy revealed a nonhomogeneous distribution of defect sites within the [Cu_3_(btc)_2−*x*_(cydc)_*x*_] crystals, with the CYDC linker incorporated into defect‐rich or defect‐free areas of selected crystals. Additionally, advanced bulk techniques have shed light on the nature of the copper species, which is highly dynamic and directly affects the reactivity of the copper sites, as shown by probe molecule FTIR spectroscopy. Furthermore, electron microscopy revealed the effect of co‐crystallizing CYDC and BTC on the crystal size and the formation of mesopores, further corroborated by X‐ray scattering analysis. In this way we have demonstrated the necessity of utilizing micro‐spectroscopy along with a whole array of bulk spectroscopic techniques to fully describe multicomponent metal–organic frameworks.

## Graphical abstract

**Questioning the Suspects**! Raman micro‐spectroscopy has revealed that defective 5‐cyano‐1,3‐benzenedicarboxylate (CYDC) linkers are located only in some crystals of [Cu_3_(btc)_2−*x*_(cydc)_*x*_] (BTC=1,3,5‐benzenetricarboxylate) after co‐crystallization into defect‐engineered HKUST‐1. Raman micro‐spectroscopy in combination with other bulk techniques has shed light on the effects of this dispersion on the material (e.g., on metal oxidation state, the formation of mesopores or the crystal size).

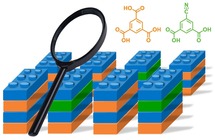

## Introduction

Metal–organic frameworks (MOFs) are an important class of porous crystalline materials consisting of organic linkers coordinated to metal atoms or clusters in a 3D fashion, which potentially leads to the presence of cavities.^1^ MOFs are industrially employed in gas storage^2^ and are potential candidates for use in other applications, such as catalysis,^3^ chemical sensing,^4^ and biomedicine.^5^ One of the advantages of MOFs compared with other porous crystalline materials, such as zeolites and mesoporous ordered aluminosilicates, is the tunability of their building blocks. Furthermore, it is possible to obtain the same topology with functionalized analogues of a given organic linker, the so‐called multivariate (MTV) MOFs.^6^ Moreover, to enhance performance in the aforementioned applications, it has been demonstrated that some properties, for example, electronic, textural, or magnetic, can be tuned by the purposeful introduction of defects within the lattice.^7^ The two main methods used for the preparation of defect‐containing MOFs are 1) post‐synthetic treatments, such as partial hydrolysis or pyrolysis of the network, and 2) the de novo synthesis of defective MOFs. In the case of the latter, the co‐crystallization of defects, that is, non‐ or weakly coordinating linker molecules,^8^ along with the putative parent linker for the topology of choice leads to a mixed‐linker framework with defect sites.^9^ Thus, the missing carboxylate, imidazolate, or phosphonate fragments in the defect linker generate more exposed cationic metallic centers. Consequently, the redox properties of the coordinatively unsaturated sites (CUSs), if already present, can be altered (e.g., reduced oxidation states).^10^ Representative examples of this strategy for the HKUST‐1 topology (a MOF consisting of copper with 1,3,5‐benzenetricarboxylate linkers) have been reported by Baiker and Fischer and their co‐workers,^8^, ^11^ in which the presence of mixed‐valence paddle‐wheel units with Cu^2+^/Cu^+^ centers were observed. Indeed, Fang et al. showed that [Cu_3_(btc)_2_] (BTC=1,3,5‐benzenetricarboxylate) can be doped with different concentrations of low‐coordinating linkers,^12^ such as 5‐cyano‐1,3‐benzenedicarboxylate (CYDC), which leads to a particular variety of MTV‐MOF in which additional Cu^+^ sites and mesopores are formed as compared with the parent, undoped [Cu_3_(btc)_2_] material. This has also been shown to be the case for its ruthenium analogue,^11^, ^13^ with which very active olefin hydrogenation and dimerization catalysts could be synthesized.9b

An indirect method to determine the spatial distribution of linkers was reported by Kong et al., who showed that a combination of solid‐state NMR spectroscopy combined with computational calculations can map differently tagged linkers in the 3D lattice.^14^ In the first example of chemical imaging of MTV‐MOFs, Katzenmeyer et al. used photothermally induced IR (PTIR) spectroscopy coupled with atom force microscopy (AFM)^15^ to prove that the aminoterephthalate and terephthalate linkers aggregate in MIL‐68(In) crystals. More recently, Schrimpf et al.^16^ used fluorescence lifetime imaging (FLIM) of tagged UiO‐67 crystals to study the distance between these labeled linkers. Most recently, Liu et al.^17^ showed by means of high‐resolution transmission electron microscopy (HRTEM) how ordered defect regions in UiO‐66 crystals form mesopores and change the overall symmetry of the lattice. Each of the above studies demonstrate the challenges associated with determining the structure of defects within defect‐engineered MOFs (DEMOFs). One of the reasons for these challenges is the difficulty of having suitable characterization methods to directly probe the local structures within DEMOF crystals. This is the topic of this article. We show that Raman micro‐spectroscopy,^18^ together with several advanced bulk characterization techniques, can be used to unravel some of the key features of linker distribution and relevant physicochemical properties, respectively, of MOFs by using [Cu_3_(btc)_2−*x*_(cydc)_*x*_] as a showcase. In addition to Raman micro‐spectroscopy, we used a vast array of other techniques to systematically assess the variation in the properties of crystals of [Cu_3_(btc)_2−*x*_(cydc)_*x*_].

## Results and Discussion

### Effect of the defect linker on mesopore formation and crystallite size of [Cu_3_(btc)_2_]

The characterization study centers around [Cu_3_(btc)_2−*x*_(cydc)_*x*_] crystals with theoretical defect concentrations *x=*0, 0.2, 0.6, 1.0, and 1.4. These crystals were prepared according to previously reported procedures (see Sections 1 and 2 in the Supporting Information).^12^

**Figure 1:**
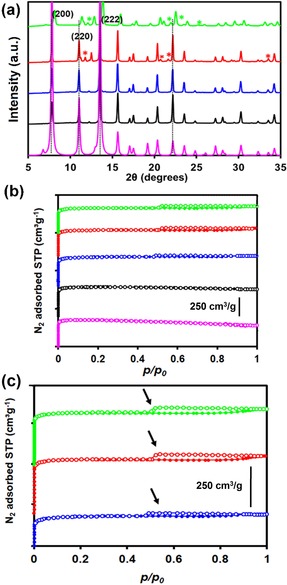
a) Simulated PXRD patterns of the parent [Cu_3_(btc)_2_] that is, *x=*0 (pink), and [Cu_3_(btc)_2−*x*_(cydc)_*x*_] materials with *x=*0.2 (black), 0.6 (blue), 1.0 (red), and 1.4 (green). The asterisks (*) correspond to different impurities present at high CYDC concentrations. b) Stacked plot of the nitrogen adsorption isotherms at 77 K of the defect‐engineered MOFs with increasing concentration of CYDC: 0 (pink), 10 (black), 30 (blue), 50 (red), and 70 mol% (green). c) Enlargement of part b) along the *y* axis to show the hysteresis loops of type H4. The arrows indicate the limit of each loop. The lack of a hysteresis loop in the samples with 0 and 10 mol% CYDC indicates the presence of mostly micropores (i.e., the absence of mesopores).

The linker composition was quantified as described by Fang et al. by means of HPLC analysis (see Section 1 in the Supporting Information) of the dissolved frameworks.^12^ Powder X‐ray diffraction (PXRD) analysis of the materials (Figure 1 a) under study showed a constant lattice parameter *d* for CYDC concentrations up to *x=*0.6, although impurities were present at this and higher loadings of the defective linker (see Figure S4). This suggests that an alternative [Cu_*x*_(cydc)_*y*_] coordination polymer phase is formed during crystallization and results in the additional peaks seen in the diffraction patterns (see Figure S5). The N_2_ adsorption isotherms recorded at 77 K (Figures 1 b,c) reveal hysteresis loops of type H4 according to the IUPAC classification, which are often associated with narrow pore slits.^24^ Nonlocal density functional theory (NLDFT) calculations of the pore size distributions with a slit geometry for an oxidic surface showed the presence of mesopores of around 13 nm when the [Cu_3_(btc)_2−*x*_(cydc)_*x*_] crystal contained 30 mol% of CYDC (Figure 1 b). Larger mesopores in the range of 15–25 nm were formed when a larger fraction of the defective CYDC linker (i.e., 50 and 70 mol% of CYDC) was present in the [Cu_3_(btc)_2−*x*_(cydc)_*x*_] crystal (see Figure S11 and Table 1).

**Table 1:** Evolution of the BET specific surface area (SSA_BET_), total pore volume (*V*_p_), *t*‐plot micropore volume (micro‐*V*_p_), and mesopore volume fractions (meso‐*V*_p_), calculated from the measured nitrogen adsorption isotherms at 77 K, with concentration of CYDC linker in the lattice.

CYDC [mol %]	SSA_BET_ [m^2^ g^−1^]	Total *V*_p_ [cm^3^ g^−1^]^[a]^	*t*‐plot micro‐*V*_p_ [cm^3^ g^−1^]	Meso‐*V*_p_ [%]^[b]^
0	1470	0.57	0.57	0
10	1592	0.58	0.58	0
30	1550	0.61	0.55	10
50	1372	0.56	0.49	12.5
70	1331	0.55	0.47	14.5

[a] Total *V*_p_ at *p*/*p*_0_=0.99. [b] Meso‐*V*_p_ as a percentage of the total *V*_p_.

**Figure 2:**
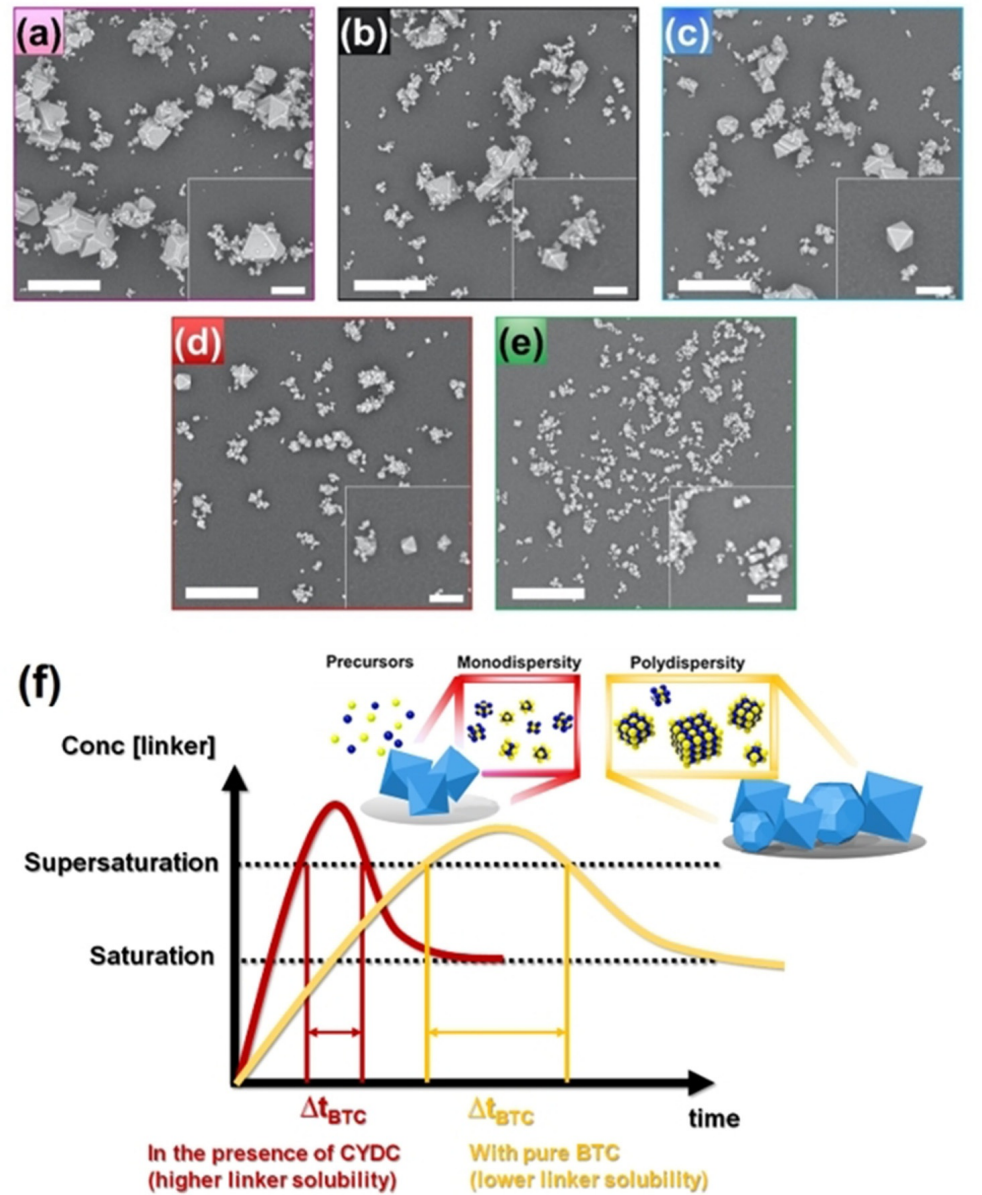
SEM images of [Cu_3_(btc)_2−*x*_(cydc)_*x*_] crystals prepared with the following compositions: a) [Cu_3_(btc)_2_], b) [Cu_3_(btc)_1.8_(cydc)_0.2_], c) [Cu_3_(btc)_1.6_(cydc)_0.4_], d) [Cu_3_(btc)_1.0_(cydc)_1.0_], and e) [Cu_3_(btc)_0.6_(cydc)_1.4_]. The insets show individual crystals with less intergrown domains. Scale bars represent 10 μm (and 5 μm in the insets). See the crystal size distributions of each material in the Supporting Information. f) LaMer diagram showing the difference in time evolution of solution concentration during crystallization for the mono‐ (including CYDC) and polydisperse (purely BTC) scenarios.

Not only porosity, but also other properties of MOF materials can be altered by different (defective) linkers. It is well established that the presence of additional synthetic agents (e.g., modulators, surfactants)9a in solution greatly affects the properties of a given MOF. In fact, the use of modulators in crystal and defect engineering has been extensively studied in the past. In particular, Kitagawa and co‐workers^25^ as well as other research groups^26^ revealed how monodentate carboxylic acids, among many other factors, such as the presence of ions^27^ or surfactants^28^ in solution, pH,^29^ solvent, ultrasound,^30^ or even acoustic waves,^31^ can steer the kinetics of the growth of either the {100} or {111} facet in HKUST‐1 with a subsequent change in the morphology from cubic to cuboctahedral and eventually to octahedral. In this study, to understand the effect on morphology of co‐crystallizing the BTC and CYDC ligands under solvothermal conditions, we performed scanning electron microscopy (SEM) on the materials synthesized. The insets in the micrographs presented in Figure 2 a–d reveal that less secondary nucleation (on the growing crystal surfaces) occurs for certain CYDC linker concentrations, leading to well‐defined octahedra and indicating that in most cases the concentration is above the critical supersaturation concentration (*c*_ss_).^32^

It is also clear that the parent [Cu_3_(btc)_2_] crystals appear as truncated octahedra, which suggests that the growth rate (*ν*) of both faces is similar, that is, *ν*_{100}_≈*ν*_{111}_. As the concentration of the defective CYDC linker increases, the morphology evolves to well‐defined octahedra, as the growth rate is much higher for the {111} facet than for {100}, that is, *ν*_{100}_≫*ν*_{111}_. Thus, we hypothesize that the defective CYDC linker hinders the growth of the {100} facet, although more detailed experimental and theoretical studies of the molecular species formed in the first stages would be necessary. Not only the crystal morphology, but also the size is affected by the presence of the defective CYDC ligand. For the parent material [Cu_3_(btc)_2_] (Figure 2 a), two large crystals of around 10–20 μm and one crystal of very small size (ca. 200–500 nm) can be seen. In contrast, when CYDC was added as the defect ligand (Figure 2 b–e), the average size decreased significantly, as can be seen in the estimated crystal size distributions shown in Figure S13 in the Supporting Information.

Moreover, the crystal size distribution is much narrower when a higher amount of CYDC is present, which led to a reduction of crystal size from 10–20 μm (10 mol% CYDC in mixture) to nearly monodisperse crystals of around 2 μm for the highest concentration of CYDC (i.e.,70 mol% CYDC). In the crystallization of HKUST‐1, analysis using the Avrami–Erofe'ev model^33^ in combination with the Sharp–Hancock^34^ method showed that under preparative synthesis conditions, nucleation continues during growth and is the rate‐limiting step.^35^ This leads to the polydispersity of crystallite sizes. However, CYDC as defective linker appears to limit crystal growth and to promote nucleation, leading to a large number of small octahedral crystals. To rule out the possibility that the CYDC linker alters the pH, we calculated the distribution of acid–base conjugates. The results are shown in Figure S12 a,b in the Supporting Information. It could be seen that similar types of protonated molecules were present at different pH values. This suggests a similar reactivity at the molecular level, in which case the CYDC would act as a capping agent hindering growth rather than modifying the acidity of the solution. Another possibility is related to the increased solubility of the CYDC linker in the solvent mixture. In this case, following LaMer's model,^36^ a higher solubility of the CYDC linker would drive the system to a faster supersaturation and hence to overcoming the activation energy for the nucleation burst sooner than in the case of BTC. Thus, a mixture containing CYDC as linker would initially yield more nuclei leading to smaller DEMOF crystallites as a result. Recent examples have shown that by controlling the supersaturation of Cu^2+^ and BTC one can, indeed, fine‐tune the crystallite size^37^ or the film thickness.^38^

### Small‐angle X‐ray scattering to probe the pore structure with increasing defect linker concentration

**Figure 3:**
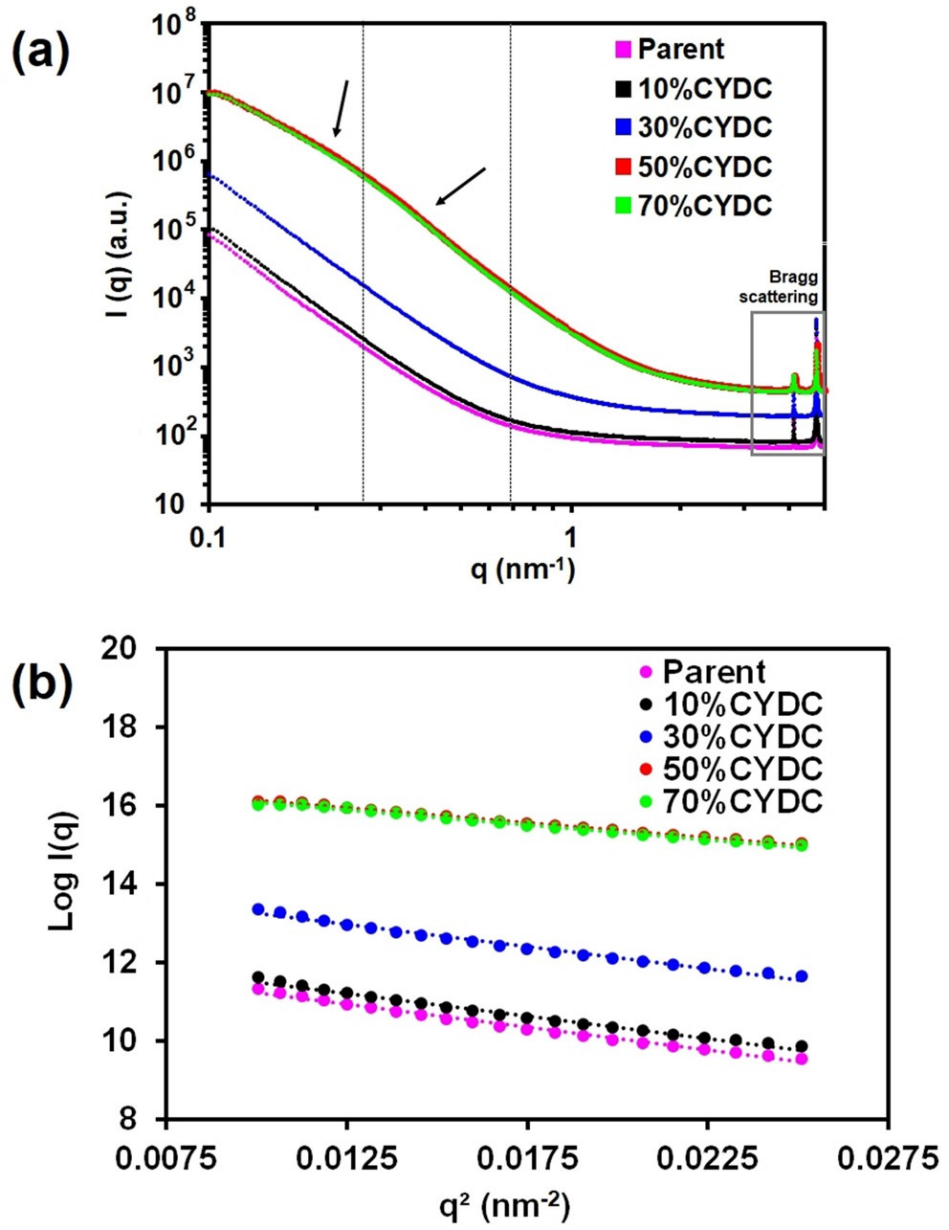
a) Small‐angle X‐ray scattering (SAXS) curves of the four defect‐engineered [Cu_3_(btc)_2−*x*_(cydc)_*x*_] powders under study (*x=*0.2, 0.6, 1, and 1.4) and the reference material [Cu_3_(btc)_2_]. The dashed lines indicate the two distinct Porod regions for the materials with 50 (*x=*1) and 70 % (*x=*1.4) CYDC defective linker. b) Log *I*(*q*) versus *q* ^2^ plot showing the linear fit used to calculate the Guinier parameters.*‘*

In the second part of our study, we used small‐angle X‐ray scattering (SAXS) to investigate the series of [Cu_3_(btc)_2−*x*_(cydc)_*x*_] materials with *x=*0, 0.2, 0.6, 1, and 1.4. The data are presented in Figure 3.Theoretical studies have predicted that a lattice of [Cu_3_(btc)_2−*x*_(L)_*x*_], in which L is a noncoordinating linker, may have a certain number of mesoporous voids without compromising mechanical stability.^19^ An analysis of mesopore formation in MOFs by SAXS has previously been reported by Tsao et al.^22^ In their case, a number of assumptions on pore geometry and dispersion within the MOF matrix made it possible to obtain model‐dependent parameters such as form factor. In this work, we also used SAXS to investigate the presence of mesopores in the different crystals under study. In our case, the structure of the mesopores remains unknown, so the analysis of the obtained SAXS data was limited to the fitting of Guinier and Porod regions. In Figure 3, the log plot of *I*(*q*) versus *q* shows large differences in the both regions with increasing concentration of CYDC (*q*>0.03 nm^−1^). By extrapolating the SAXS data to *q*=0 using an approximation for single particle scattering yields Equation (1), 23

(1) $$I\left(q\right)=I\left(0\right)e^{-\left[\frac{{\left(qR_{g}\right)}^{2}}{3}\right]}$$

in which *I*(0) represents a contrast parameter, the excess of electrons of the scatterer relative to its surroundings, and *R*_g_ is the radius of gyration, which is indicative of the size of the scatterer.

As can be seen in Figure 3 and Table 2, the higher values of *I*(*q*) in the *q*>0.03 nm^−1^ region with increasing CYDC content can be correlated with less dense materials, that is, more scattered X‐rays, due to the presence of more (or larger) mesopores. The presence of those mesopores is corroborated by the two distinct regions at higher *q* values (*q*>0.2 nm^−1^) for the materials with high loadings of CYDC, that is, 50 and 70 mol%. Porod's law describes the scattering at higher *q* values according to Equation (2)

(2) $$I\left(q\right)=Kq^{-D}$$

**Table 2:** Overview of the values of the different parameters obtained by fitting the Guinier–Porod regions of the SAXS data of the defect‐engineered [Cu_3_(btc)_2−*x*_(cydc)_*x*_] powders.

CYDC [mol%]^[a]^	*I*(0)^[a]^	*R_g_* [nm]^[a,b]^	*D*_1_^[c]^	*D*_2_^[d]^
0	0.311×10^6^	20.2(±0.4)	4.5	–
10	0.354×10^6^	19.5(±0.4)	4.04	–
30	2.15×10^6^	19.3(±0.4)	4.03	–
50	24.2×10^6^	15.14(±0.2)	2.75	4.18
70	23.9×10^6^	15.6(±0.2)	2.86	4.14

[a] Calculated by using the Guinier equation in the range 0.01<*q*<0.02 nm^2^. [b] Values in parentheses indicate the relative error in the *R_g_* value. [c] Calculated by using the Porod equation in the range 0.14<*q*<0.2 nm^−1^. [d] Calculated by using the Porod equation in the range 0.27<*q*<0.7 nm^−1^.

in which *K* is a constant and the scattering depends on the exponential value *D*. In line with literature data, the fitting of the scattering measured for the MOFs with 50 and 70 mol% of the defective CYDC linker in the 0.14<*q*<0.2 nm^−1^ region yielded exponential values of around −2.8. At higher *q*, this value approached the typical *D=*4, as it did for materials with lower concentrations of the defective CYDC linker, corroborating the lack of mesopores within those materials. The differences in slope are typically associated with the presence of a higher polydispersity of the scatterer volumes and confirm the presence of pores with different radii, that is, micropores of 13.2, 11.1 and 5 Å diameter, in addition to the potential mesopores generated by CYDC.

The above data indicate that there are mainly three types of DEMOF materials, as can be distinguished by our SAXS data: 1) Materials with no defects or a negligible effect on the pore structure (0 and 10 mol% of CYDC), 2) materials with intermediate mesopores (30 mol% of CYDC), and 3) materials with large cavities within the MOF (50 and 70 mol% of CYDC).

**Figure 4:**
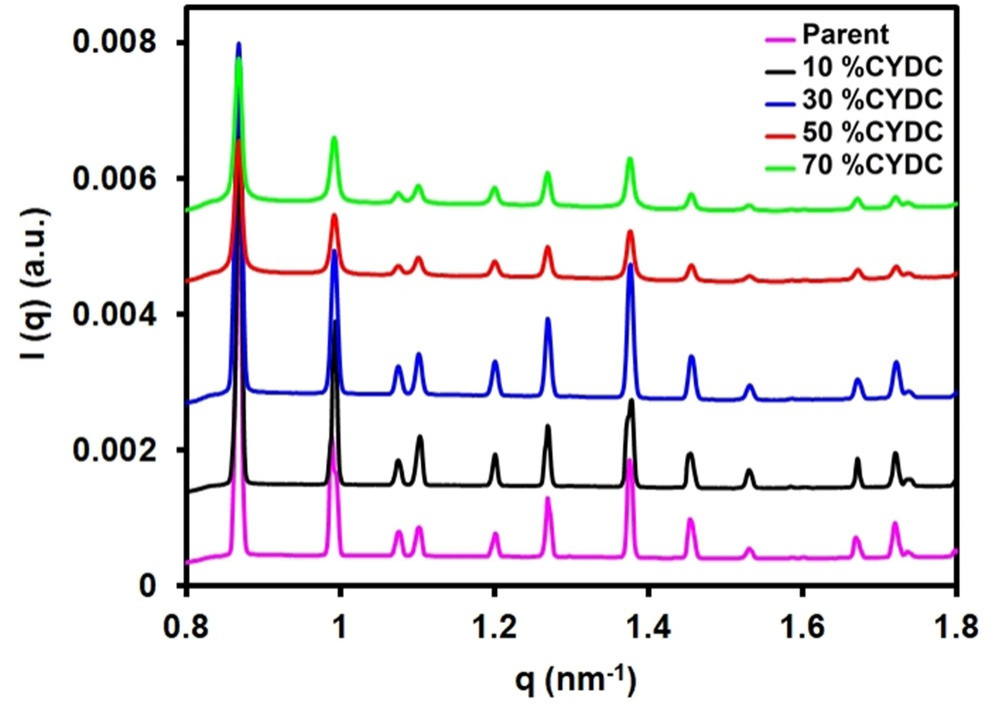
WAXS profiles of the defect‐engineered materials with increasing concentration of CYDC showing the Bragg reflections indicative of [Cu_3_(btc)_2_] at all concentrations: [Cu_3_(btc)_2_] (pink), [Cu_3_(btc)_1.8_(cydc)_0.2_] (black), [Cu_3_(btc)_1.6_(cydc)_0.4_] (blue), [Cu_3_(btc)_1.0_(cydc)_1.0_] (red), and [Cu_3_(btc)_0.6_(cydc)_1.4_] (green).

The long‐range crystallinity, as shown in the PXRD patterns, was further corroborated by collecting the wide‐angle X‐ray scattering (WAXS) patterns of the different materials under study (see Figure 4). These observations corroborate the findings made with SAXS, PXRD as well as Raman micro‐spectroscopy (see below).

### Chemical imaging of the CYDC spatial distribution by Raman micro‐spectroscopy

**Figure 5:**
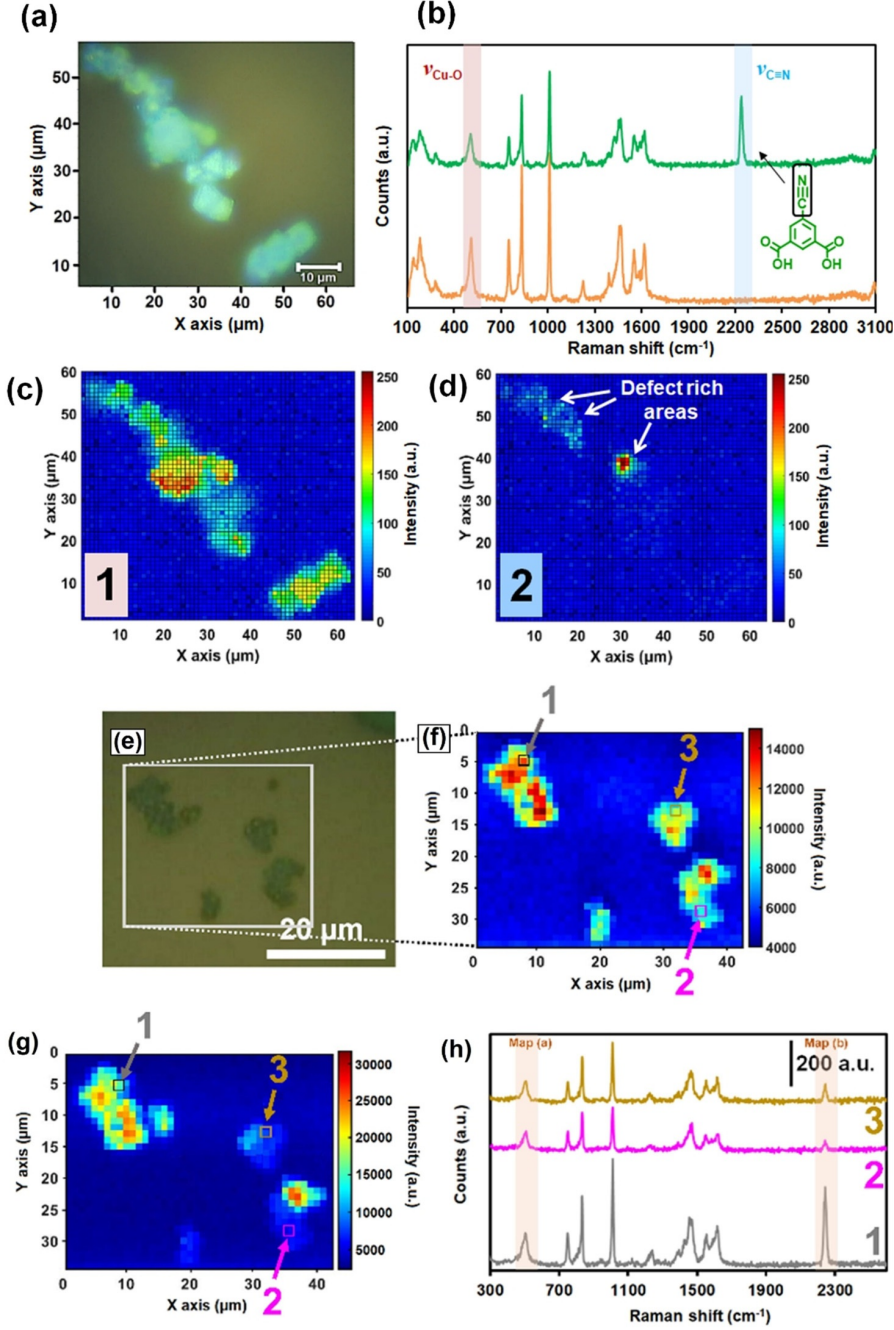
a) Optical image of a set of chemically mapped [Cu_3_(btc)_1.0_(cydc)_1.0_] crystals. b) Raman spectra of two selected pixels in which the defective linker, that is, CYDC (green), and the parent linker, that is, BTC (orange), can be observed. Signal‐to‐baseline maps of the Raman bands corresponding to c) the Cu−O stretching vibration at 505 cm^−1^ (480–520 cm^−1^ region) and d) the C≡N stretching mode at 2240 cm^−1^ (2220–2260 cm^−1^ region). The white arrows show the defect‐rich areas of the selected [Cu_3_(btc)_1.0_(cydc)_1.0_] crystals. e) Optical image of several [Cu_3_(btc)_0.6_(cydc)_1.4_] crystals with the corresponding Raman micro‐spectroscopy maps of f) the 480–520 cm^−1^ and g) 2220–2260 cm^−1^ spectral regions. h) Raman spectra of selected, exemplary pixels in f) and g) showing different contents of the CYDC linker and [Cu_3_(btc)_2_] material. Note that the different areas show a low intensity of the ν(C≡N) Raman stretching mode at 2240 cm^−1^ corresponding to a heterogeneous distribution of the defect linker CYDC. The intensities of the maps are signal‐to‐baseline normalized.

To study the distribution of CYDC in different crystals, we made use of Raman micro‐spectroscopy (Figure 5). The obtained Raman micro‐spectroscopy data clearly show the presence of interparticle heterogeneities rather than a homogeneous distribution of the defective linker (CYDC) throughout the different crystals. Here, a set of defective MOF crystals with the theoretical composition [Cu_3_(btc)_1.0_(cydc)_1.0_], drop‐cast from a CH_2_Cl_2_ suspension onto a glass cover, was imaged. The optical image in Figure 5 a demonstrates that the CYDC linker is present only in selected areas of the DEMOF crystals. Figure 5 b shows two exemplary Raman spectra recorded for 1) a pixel in which the Raman band corresponding to the stretching mode of the C≡N bond at 2241 cm^−1^ can be seen (green), and thus the defective linker CYDC is visible, and 2) a pixel in which this Raman band is not present and only the Raman bands associated with the [Cu_3_(btc)_2_] structure are observable (orange). The ν(C≡N) Raman band is observed at 2247 cm^−1^ in pure CYDC (see Figure S14b in the Supporting Information), which is a blueshift (Δ*ν*) of around 6 cm^−1^ compared with the CYDC embedded in the DEMOF lattice. This may be ascribed to the partial donation of the nitrogen atom in the CN group to the copper cations in the paddle‐wheel structure, or to vibrational restrictions imposed by the lattice structure itself. However, the observed band shift also suggests that the CYDC linker is, indeed, part of the crystal structure and not simply physically deposited within the pores of the HKUST‐1 structure. However, the nature of the defects themselves (e.g., defective paddle wheels and mesopores) within the lattice is still under debate,^19^ thereby making it a complicated task to elucidate the underlying reason for this observed band shift in the Raman spectra. Representative signal‐to‐baseline ratio maps of the region corresponding to the ν(Cu−O) stretching mode at 505 cm^−1^ (Figure 5 c, Map 1) and that of the stretching mode of the ν(C≡N) band at 2241 cm^−1^ of the CYDC linker (Figure 1 d, Map 2) were obtained.

The obtained Raman micro‐spectroscopy maps of the [Cu_3_(btc)_0.6_(cydc)_1.4_] crystal exhibit similar phenomena, with a higher number of pixels in which the ν(C≡N) Raman band is observed (Figure 5 f,g). Three locations have been selected to showcase the differences observed in the Raman spectra.

Pixel 1 (gray color) shows a high intensity of both the fingerprint bands corresponding to [Cu_3_(btc)_2_]^20^ and the ν(C≡N) Raman band. The count ratio ν(C≡N)/ν(Cu−O) was *I*_C≡N_/*I*_Cu−O_=1.66 for the Raman spectrum measured in pixel 1, which indicates the presence of a large number of C≡N bonds, and thus of the defective linker CYDC. In contrast, the *I*_C≡N_/*I*_Cu−O_ ratio was 0.75 and 0.99 for the Raman spectra collected in pixels 2 and 3, respectively. It is worth mentioning that the differences in Raman intensity may arise when using a laser, given that the probed objects might be at different focal planes.

The Raman micro‐spectroscopy data of the crystals with lower content of CYDC, namely [Cu_3_(btc)_1.4_(cydc)_0.6_] and [Cu_3_(btc)_1.8_(cydc)_0.2_], reveal the lack of observable defect‐rich regions (see Figure S14 in the Supporting Information) in comparison with the other two materials (see Figures 1 and 2). This can be ascribed to 1) the low sensitivity of the Raman micro‐spectroscopy method, 2) the insufficient spatial resolution of Raman micro‐spectroscopy, and 3) the absence of defect‐rich regions. Moreover, spherical aberration and scattered light, which varies as a function of the depth to which the incident beam is focused on the crystals, could also have an impact. However, for the experimental configuration employed here, Everall described how these phenomena become relevant only at probing distances ≥3–5 μm,^21^ which are more than the average size of the crystals measured in this work, thereby suggesting that the differences in Raman intensity are not associated with spectral artefacts.

### Presence of Cu^2+^/Cu^+^ on the paddle‐wheel clusters with increasing defect linker concentration

**Figure 6:**
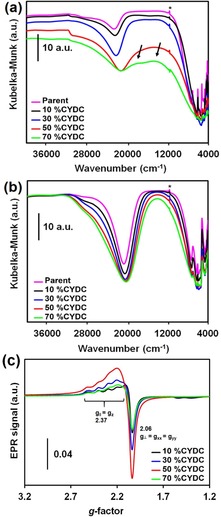
DR UV/Vis/NIR spectra of the [Cu_3_(btc)_2−*x*_(cydc)_*x*_] crystals with *x=*0 (pink), 0.2 (black), 0.6 (blue), 1.0 (red), and 1.4 (pink) a) after and b) before degassing under vacuum (*p*<1 mbar) at 423 K for 15 min. The peak indicated by * corresponds to the monochromator switch at 860 nm. The arrows in a) indicate the presence of two distinct types of d– transitions of Cu^2+^ ions in the lattice. c) EPR spectra of [Cu_3_(btc)_2−*x*_(cydc)_*x*_] crystals with *x=*10 (black), 30 (blue), 50 (red), and 70 mol% CYDC (green). The full‐width sweep showing the hyperfine coupling lines of Cu^2+^ with *S*=1/2 and its nuclear spin *I*_Cu_=3/2 becomes diluted as the concentration of the defective CYDC linker increases.

As mentioned above, the introduction of noncoordinating linkers results in the presence of Cu^+^ as well as a higher tendency towards the reduction of Cu^2+^ to Cu^+^ in the paddle‐wheel units. Thus, we have systematically studied the oxidation state of copper in the [Cu_3_(btc)_2−*x*_(cydc)_*x*_] crystals by means of diffuse reflectance (DR) UV/Vis/NIR spectroscopy. In Figure 6 a.b, the different DR UV/Vis/NIR spectra show that the absorption band at around 14 200 cm^−1^, which corresponds to Cu^2+^ with *O_h_* distorted symmetry and oxygen or OH‐like ligands, that is, the metal sites, decreases with increasing CYDC concentration in the [Cu_3_(btc)_2−*x*_(cydc)_*x*_] materials, as expected for the reduction of Cu^2+^ to Cu^+^. Accordingly, the ligand‐to‐metal charge‐transfer (LMCT) band at around 33 333 cm^−1^ decreases in intensity with increasing content of the CYDC linker, in accord with the spectrum of pure 5‐cyano‐1,3‐benzenedicarboxylic acid (see Figure S14 in the Supporting Information), which shows a sharper π→π* excitation band than 1,3,5‐benzenetricarboxylic acid.^39^

To exclude the effects of solvent in the pores, the materials were degassed under vacuum at 423 K for a short period of time (only 5 min, to avoid Cu^2+^ autoreduction as much as possible).^40^ The DR UV/Vis/NIR spectra show a shoulder at around 25 000 cm^−1^, which corresponds to a change in the symmetry of the copper centers upon loss of axial water. The intensity of this shoulder decreases with higher content of the CYDC linker in the [Cu_3_(btc)_2−*x*_(cydc)_*x*_] crystals. At higher concentrations of the CYDC linker (*x=*1.4), both spectral features, that is, the Cu^2+^ d–d transition and the high‐energy shoulder at around 25 000 cm^−1^, are distinguishable as different absorption bands. This indicates two effects induced by the presence of the defective CYDC linker: 1) Even before activation, a certain fraction of copper is already in the Cu^+^ state and 2) degassing leads to large fractions of Cu^+^ in the framework.

To further study the nature of the copper species present in the [Cu_3_(btc)_2−*x*_(cydc)_*x*_] materials, we recorded X‐band continuous wave (CW) EPR spectra at 100 K. Pöppl and co‐workers have previously shown a multitude of copper species within [Cu_3_(btc)_2_] (including Cu^2+^ dimers, that is, paddle‐wheels, and extra‐framework monomeric [Cu(H_2_O)_6_]^2+^ complexes)^41^ as well as the presence of Cu^+^ sites present before activation in defect‐engineered [Cu_3_(btc)_2_] samples. Moreover, Todaro and co‐workers40c, 42 reported the presence of different types of copper sites in [Cu_3_(btc)_2_] crystals, formed by the activity of water in the air on the paddle wheels that yields both Cu^2+^ and Cu^+^ centers. In their work, they showed how the typical antiferromagnetic coupling arising from the overlapping wave functions of both d^9^ cations in Cu_2_(OR)_4_ carboxylate paddle‐wheel dimers with *S*=1/2 is not maintained when one of the cations is reduced to Cu^+^.

As demonstrated by the DR UV/Vis/NIR spectra shown in Figure 5 a,b, the CW EPR spectra also reveal that the frameworks with increasing amounts of CYDC have higher amounts of Cu^+^ (Figure 6 c and Table S4 in the Supporting Information). This can be seen in the less‐defined hyperfine lines corresponding to the hyperfine quadruple (*S*=1/2 ) in *g*_*‖*_ as the loading of the CYDC linker increased. It is interesting to highlight that the highest concentration of Cu^+^ appears to be present in the crystals with a loading of 50 mol% CYDC. This may be ascribed to the formation of a [Cu_*x*_(cydc)_*y*_] coordination polymer or extra‐framework Cu^2+^ cations when higher concentrations of the defective linker are used, rather than to co‐crystallization in the MOF lattice as defects.

### Redox pairs and defect clusters studied by means of pyridine and NO probe FTIR spectroscopy

**Figure 7:**
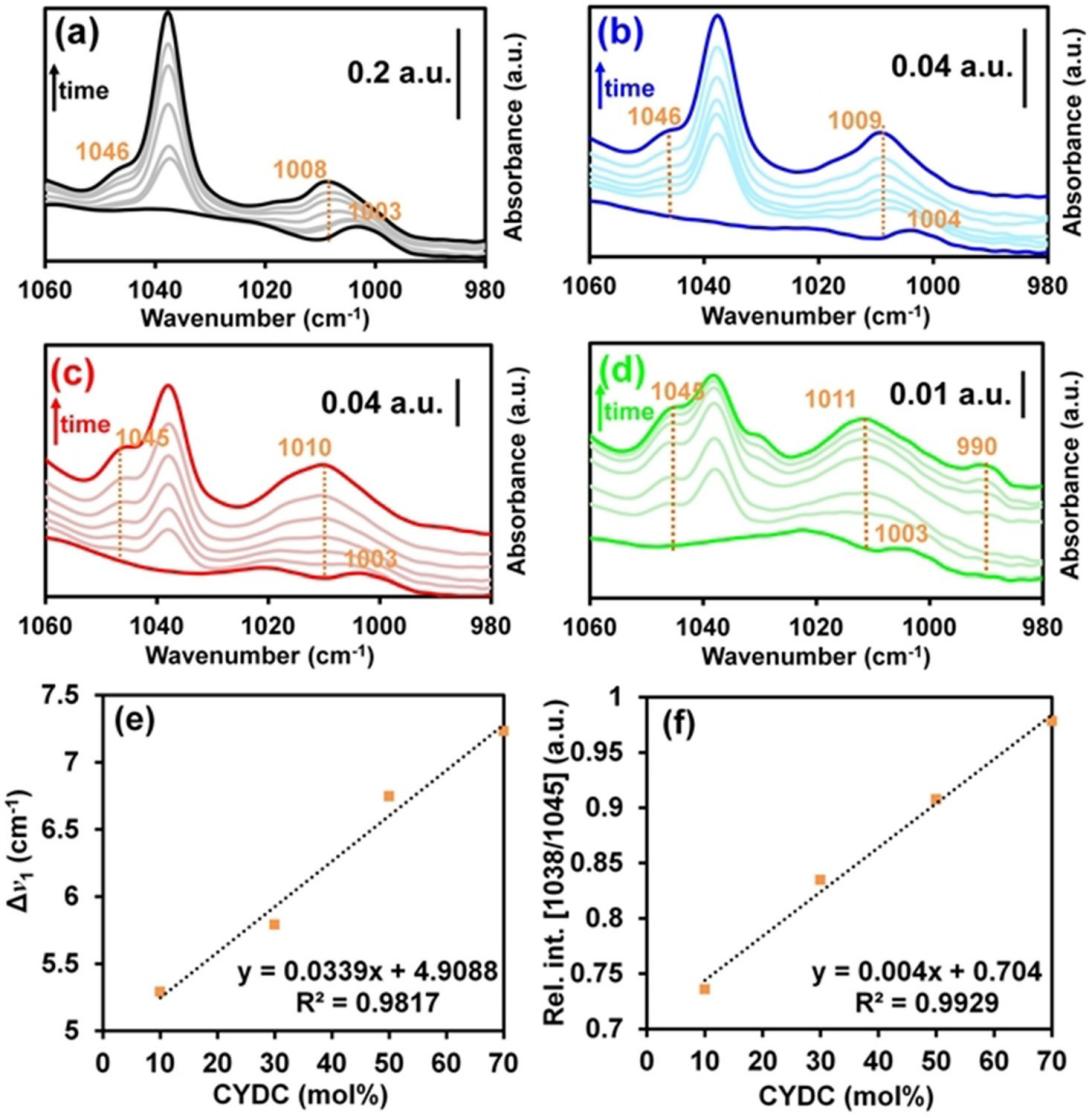
Pyridine‐probed FTIR spectra of the [Cu_3_(btc)_2−*x*_(cydc)_*x*_] crystals with *x*=0.2 (a), 0.4 (b), 1.0 (c), and 1.4 (d) showing the bands corresponding to ν_12_ and ν_1_ (ca. 1038 and 1008 cm^−1^, respectively) asymmetrical ring‐stretching modes. e) Evolution of the shift of the ν_1_ mode (band at ca. 1008 cm^−1^) with increasing CYDC concentration. f) Increase of the relative intensity of the shoulder at around 1045 cm^−1^ with increasing CYDC concentration.

To better understand the behavior of the copper sites as Lewis acids, we used pyridine (Py) as a molecular probe in combination with FTIR spectroscopy. It can be seen in Figure 7 that in addition to the ν_12_ vibrational mode of Py adsorbed onto the Lewis CUS of Cu^2+^ at around 1038 cm^−1^,^43^ a shoulder at around 1046 cm^−1^ is observed, assigned to the interaction of the σ‐donating nitrogen lone pair of Py with the defect Cu^+^ sites,^44^ in contrast to previous literature reports.^45^ The intensity of this shoulder gradually increased with increasing concentration of the defective CYDC linker in the MOF lattice, further corroborating our hypothesis. Py FTIR spectroscopy has previously been used for the characterization of acid and redox metal sites in MOF materials.^46^ However, to the best of our knowledge, up to now, no studies have been carried out with defective HKUST‐1 systems in which redox‐active Cu^2+^/Cu^+^ sites are also present.

Moreover, in the spectra of the DEMOF crystals containing 50 and 70 mol% CYDC, a shoulder develops at around 1014 cm^−1^. Previously, the formation of such a shoulder has been ascribed to the interaction of pyridine with reduced Fe^2+^/Fe^3+^ in MIL‐100(Fe) systems.46c A shoulder next to the ν_12_ band, at around 1045 cm^−1^, simultaneously develops over time for each sample, and its relative intensity (compared with the band at ca. 1037 cm^−1^) increases with increasing amounts of the defective CYDC linker in the HKUST‐1 crystals. Fang et al. demonstrated by means of CO probe FTIR spectroscopy and X‐ray photoelectron spectroscopy (XPS) that higher amounts of reduced Cu^+^ sites are present with increasing amounts of the CYDC linker.^12^ Thus, this shoulder can be correlated with pyridine interacting with the less acidic Cu^+^ sites, because ring bending appears at higher energy. This stems from a weaker N–Cu^+^ interaction (less σ‐donation than for Cu^2+^), which allows for higher‐energy flexing of the C−C and C−H bonds. In the case of the material containing 70 mol% CYDC, two additional features appeared after exposure to pyridine for 60 min, namely a shoulder at 1031 cm^−1^ and a band at 990 cm^−1^. These bands correspond to non‐coordinated pyridine gas (to the ν_1_ and ν_12_ modes, respectively),^43^ which indicates that all the Cu^*x*+^ sites are saturated with pyridine. We hypothesize that this is related to the lower surface area of this material, in which not all the copper sites are available for coordination by pyridine. Further experiments making use of larger probes, such as collidine,^47^ may help in elucidating whether the Cu^+^ sites are indeed present inside the pores or on the external surface of the [Cu_3_(btc)_2−*x*_(cydc)_*x*_] crystals.

**Figure 8:**
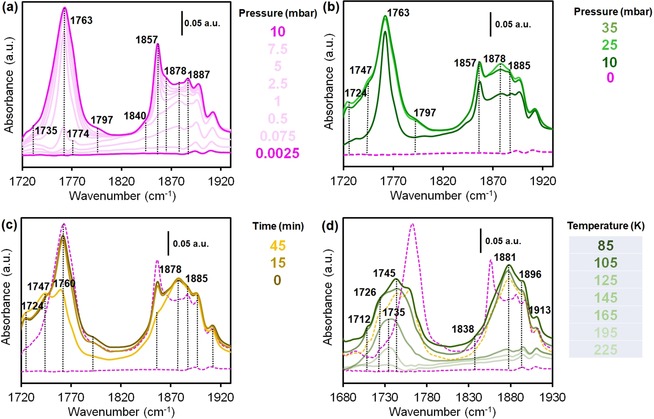
FTIR spectra of parent HKUST‐1: a) After dosing with the 10 % NO/He v/v pressures indicated at 85 K, b) at high NO pressures, up to 35 mbar (the dashed line represents the clean cell) at 85 K, c) the desorption of NO at constant temperature at *p*<10^−5^ mbar over time at 85 K, and d) at increasing temperature (the dashed pink lines correspond to previous spectra recorded at 0 and 10 mbar). The pellet was activated under *p*<10^−5^ mbar (pink dashed line with no bands) at 298 K for 24 h and further at 423 K (5 K min^−1^) for 5 min (to avoid heat‐induced reduction).

To compare the behavior of the defect sites towards reactive probes, FTIR spectroscopy in combination with NO as a probe was used. The spectra of the parent [Cu_3_(btc)_2_] material under different NO pressures and temperatures are shown in Figure 8. It can be seen that two large sets of bands at 1720–1800 and 1840–1920 cm^−1^ develop with increasing NO pressure. This contrasts with previous reports, in which only a strong band was observed, although different evacuation procedures were used.40a However, FTIR spectra similar to those described here have previously been observed for copper‐exchanged zeolites.^48^ A very intense, broad band centered at around 1763 cm^−1^, ascribed to Cu^+^⋅⋅⋅(NO)_2_ dimers, appears at low temperature and became more intense at *p*_NO_>1 mbar. This band shows similar behavior to the one at 1857 cm^−1^. A low intensity band at around 1774 cm^−1^ may correspond to either the *trans* isomers of Cu^+^⋅⋅⋅(NO)_2_ species, or to (NO)Cu^+^(NO) species.^49^ Despite its high intensity, which suggests a high number of Cu^+^ sites, a high molar extinction coefficient has been reported for adsorbates interacting with Cu^+^ sites.^50^ A small band at around 1735 cm^−1^ appears at low pressures, as well as a shoulder at around 1797 cm^−1^. These bands are assigned to different reduced Cu^+^ species with a slightly different chemical nature to that of the paddle‐wheel metal atoms, for example, defects and extra‐framework copper cations. On the other hand, the band at around 1881 cm^−1^ has previously been assigned to Cu^2+^⋅⋅⋅NO species, which remain strongly adsorbed after desorption over time, the band decreasing in intensity only at *T*>200 K. An overlapping band at around 1787 cm^−1^ showing very similar behavior is assigned to slightly different Cu^2+^ sites. It has previously been reported that up to around 40 % of copper in HKUST‐1 may be reduced^51^ due to the presence of different types of paddle‐wheel (Cu^2+^/Cu^+^ or Cu^2+^/Cu^2+^) units, and this might explain the presence of this band. Two small features at around 1896 and 1913 cm^−1^ are present before the introduction of NO, and have been previously assigned to combination bands of the aromatic backbone.40a, 52

**Figure 9:**
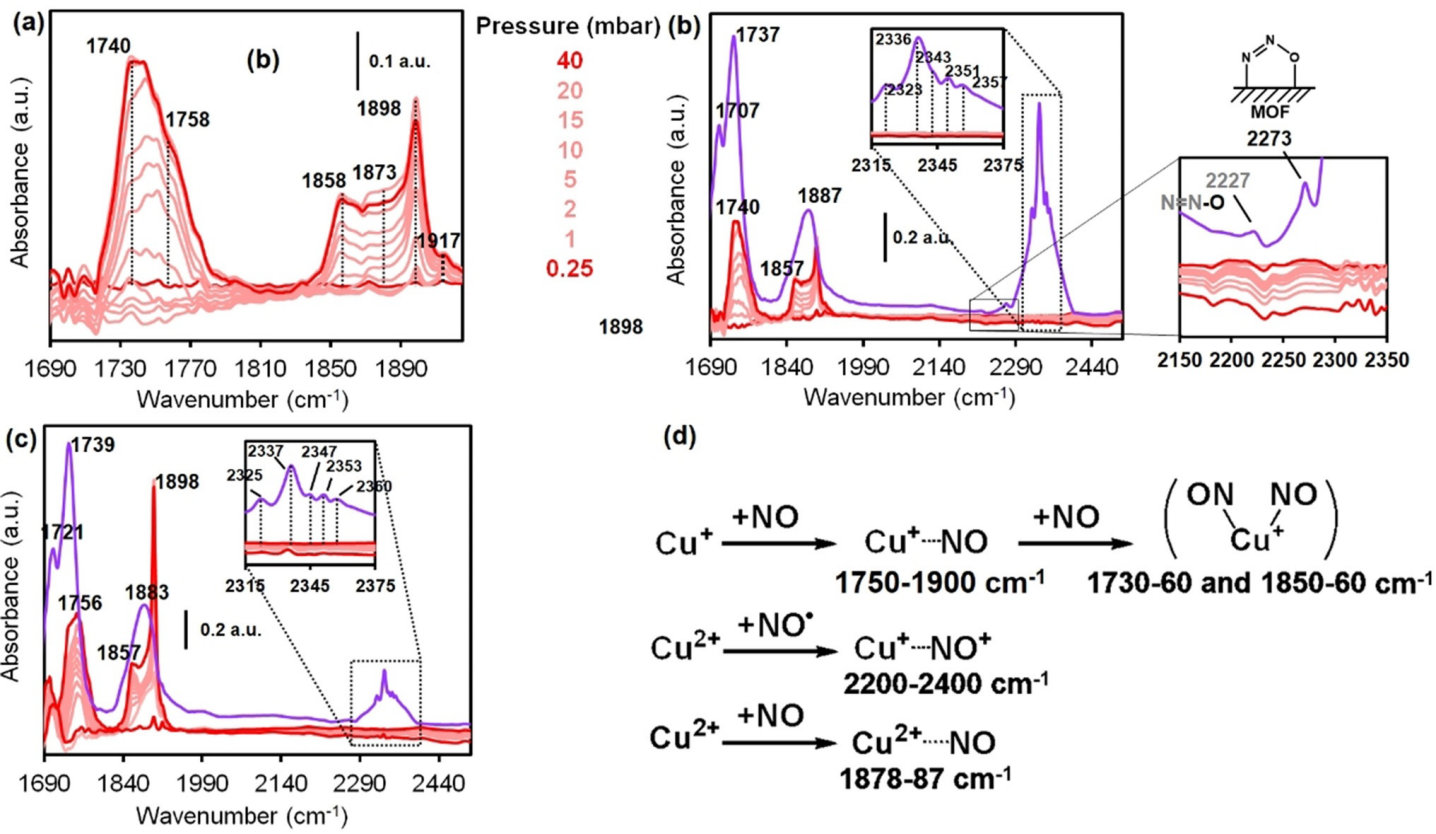
FTIR spectra of [Cu_3_(btc)_2−*x*_(cydc)_*x*_] crystals with *x=*1.0 at 85 K. a) Stepwise dosing of NO to the equilibrium pressures noted. b) Saturation of the cell with NO after 5 min. c) Experiments with identical pressures but with a heating pretreatment of 16 h. For a) and b), the pellet was activated under *p*<10^−5^ mbar at 298 K for 24 h and further at 423 K (5 K min^−1^) for 5 min (to avoid heat‐induced reduction). For c), the second treatment was carried out for 16 h instead of 5 min. d) Scheme for the adsorption of NO on the copper sites, as described in ref. 54.

In contrast, Figure 9 a–c shows that, although a similar profile with two sets of bands is seen for the [Cu_3_(btc)_1.0_(cydc)_1.0_] material, a number of differences are evident. First, the material, upon activation under similar conditions to the parent HKUST‐1 (RT for 16 h, then 5 min at 423 K), showed a redshift of 17 cm^−1^ of the band corresponding to the ν_as_(N−O) mode of the Cu^+^⋅⋅⋅(NO)_2_ dimer. This was not observed for the symmetric mode ν_s_(N−O), which appears at around 1857 cm^−1^, as for the parent HKUST‐1. Moreover, the shape of the band looks rather asymmetric, which suggests that another band of lower intensity at around 1758 cm^−1^, corresponding to Cu^+^⋅⋅⋅NO adsorbates in different types of copper sites, may be overlapping. The bands at around 1898 and 1917 cm^−1^ corresponding to the framework are strongly affected by the dosing of NO, especially the former one. The intensity of this band is much higher after longer thermal pretreatment, which indicates that the activation procedure affects not only the metal CUS sites, but also the organic backbone. At higher energies, around 2200–2400 cm^−1^, a set of bands are visible that are typically assigned to N_2_, NO_2_, and N_2_O, which indicates that similar pretreatments affect the fraction of reduced Cu^+^ sites, as well as their reactivity, in a different manner.

A number of mechanisms for the adsorption of NO have been described for zeolite materials loaded with copper, for example, Cu‐ZSM‐5, that may be applicable to this case.^54^ Others argue that the chemistry is more related to that of copper‐containing enzymes due to the single‐site character of the copper sites.^55^ In any case, the results of this study clearly show that different surface N_*x*_O_*y*_ species are formed depending on the pretreatment. Although the spectral profile is similar, the maximum of the band corresponding to the Cu^+^⋅⋅⋅NO adducts is slightly blue‐shifted from around 1740 to around 1756 cm^−1^, which suggests that the species present after pretreatment for 5 min are different to those formed after 16 h of heating at 423 K.

In summary, we have found that both the amount of CYDC and the pretreatment conditions greatly affect the nature of the copper sites in the materials. Experiments with other nonreactive probes for Lewis acids, for example, CD_3_CN, may be beneficial for better determining the properties of these copper sites.

## Conclusions

The spatial distribution of two different non‐defective and defective linkers, namely 1,3,5‐benzenetricarboxylate and 5‐cyano‐1,3‐benzenedicarboxylate, respectively, in different MOF crystals of the HKUST‐1 topology has been studied. We have shown that the defective linkers (CYDC) are heterogeneously dispersed throughout the [Cu_3_(btc)_2−*x*_(cydc)_*x*_] MOF crystals and aggregate in relatively small clusters rich in defective linkers. These small clusters result in the formation of mesopores that are also heterogeneous in size and geometry. Furthermore, the introduction of these defective linkers results in a decrease in the crystallite size due to different supersaturation regimes. Additionally, we have demonstrated the presence of a higher fraction of Cu^+^ sites within the pores. These sites have different reactivities towards molecular probes such as NO. Our work highlights the importance of using a wide variety of advanced characterization tools, such as vibrational spectroscopy (Raman and FTIR with probe molecules), electronic spectroscopy (DR UV/Vis/NIR), magnetic resonance spectroscopy (EPR), X‐ray scattering and diffraction (SAXS, WAXS, and PXRD), and electron microscopy (SEM), for understanding the underlying chemistry of complex, multicomponent porous materials, including MOF materials, and provides guidelines for the improved design of defect‐engineered frameworks.

## Experimental Section

**Synthesis of [Cu_3_(btc)_2−*x*_** **(cydc)**_***x***_ **]**: Crystals of [Cu_3_(btc)_2−*x*_(cydc)_*x*_] were synthesized according to the protocol described in the literature.^12^ More details can be found in the Supporting Information.

**Methods of characterization**: Raman micro‐spectroscopy measurements were carried out with a Renishaw Raman InVia^TM^ microscope with laser excitation at 532 nm, a power density of 6×10^−3^ W cm^−2^, operated at 36 mW total power (power output=1 %), a 1200 lines mm^−1^ grating, detector slits, and a CCD detector. A silicon wafer was used as reference and samples were prepared by dispersing the crystals in dichloromethane (VWR International, 98 % CH_2_Cl_2_), dropping onto a glass cover, and measuring after the solvent had dried in air at room temperature. The crystals were then imaged with a ×50 objective and the spectra collected with a pixel size of 1×1 μm^2^, one accumulation, and 10 s per accumulation. Multiple exposures were carried out to ensure that sample damage was not observed. Data processing was performed with Wire 3.4^©^ of the TXM Wizard software package^53^ and Matlab R2015a^56^ by generating signal‐to‐baseline maps of the spectral regions described in the main text (cosmic ray signals and artefacts were manually subtracted).

PXRD patterns were obtained by using a Bruker‐AXS D2 Phaser powder X‐ray diffractometer operated at 30 kV in Bragg–Brentano geometry using Co_Kα1,2_ radiation (*λ*=1.79026 Å). Measurements were carried out between 5 and 70° in steps of 0.05° at a scan speed of 1 s. Simulated patterns were obtained by processing the corresponding .cif files using Mercury 3.7^®^ (*λ*=1.79026 Å, FWHM=0.2).

SEM images were recorded on a PhenomPro X microscope with a CsB filament operated at 10 kV. The powder samples were supported on carbon tape deposited over aluminum stabs (FEI stabs) and inserted in the microscope vacuum chamber without gold or platinum coating.

SAXS and WAXS experiments were carried out at the BM26b beamline of the Dutch/Belgian line (DUBBLE) at the European Synchrotron Radiation Facilities (ESRF) in Grenoble, France. The samples were mounted in quartz capillaries and the SAXS images were collected by using a Pilatus 1M detector (169 mm×179 mm active area), whereas the WAXS patterns were collected by using a 300K‐W linear Pilatus detector (254 mm×33.5 mm active area) using a beam energy of 1.033 Å. Data acquisition, analysis, and fitting were performed by using the FIT2D and SASfit packages.

FTIR spectra were recorded on a PerkinElmer System 2000 spectrometer (32 scans, 4 cm^−1^ resolution, DTGS detector, cell with KBr windows). The samples were prepared in a press tool by pressing around 10 mg of powder into self‐supported pellets (2 cm^2^ area), which were then activated under the conditions described in the main text. Thereafter, the cell was cooled with liquid N_2_ at a temperature down to 85 K, and then a mixture of 10 % NO/He (v/v, Linde AG, 99.9 %) was introduced stepwise into the cell through a stainless‐steel manifold and a three‐way valve to the pressures indicated in the main text. After dosing, the cell was let to warm naturally to the indicated temperatures and the spectra collected at the pressure described above. Pyridine was used as a probe molecule, similar pellets were placed in a cell similar to the one described above and the spectra recorded with a ThermoFisher Nicolet iS5 spectrometer (32 scans, 4 cm^−1^ resolution, DTGS detector). The pellet was evacuated by thermal treatment in a cell at 498 K (ramp of 10 K min^−1^) for 24 h at *p*<10^−5^ mbar and then cooled to 323 K. At that temperature, pyridine (redistilled, 99.9 %, Sigma–Aldrich) vapor was introduced into the cell and the equilibrium pressure set to 15 mbar. Spectra were recorded in adsorption mode up to 60 min after introduction of the gas.

DR UV/Vis/NIR spectra were recorded with a PerkinElmer Lambda 950 S spectrometer equipped with InGaAs and photomultiplier (PMT) detectors and D_2_ and W halogen lamps (4 nm s^−1^, 2 nm^−1^ resolution, Halon polymer reference). Powders measured in air were placed in a holder with quartz windows and the spectra were recorded immediately. In the case of activated materials, the powders were dried at 423 K under primary vacuum (*p*<1 mbar) for 15 min, then transferred into a glovebox (BraunTM, O_2_<1 ppm, H_2_O<1 ppm), subsequently placed in a sealed quartz holder, and finally the spectra were recorded.

EPR experiments were carried out in a Bruker EMX spectrometer at a frequency of about 9.5 GHz (X band). The magnetic‐field modulation frequency was 100 kHz. The temperature was set to 100 K by spraying liquid nitrogen on the quartz tubes filled with around 20 mg of each MOF sample.

## Conflict of interest

The authors declare no conflict of interest.

## Supporting information

As a service to our authors and readers, this journal provides supporting information supplied by the authors. Such materials are peer reviewed and may be re‐organized for online delivery, but are not copy‐edited or typeset. Technical support issues arising from supporting information (other than missing files) should be addressed to the authors.

Supplementary

## References

[1a] H. Li , M. Eddaoudi , M. O'Keeffe , O. M. Yaghi , Nature 1999, 402, 276–279;

[1b] H.-C. Zhou , J. R. Long , O. M. Yaghi , Chem. Rev. 2012, 112, 673–674. 22280456 10.1021/cr300014x

[2a] J.-R. Li , R. J. Kuppler , H.-C. Zhou , Chem. Soc. Rev. 2009, 38, 1477–1504; 19384449 10.1039/b802426j

[2b] T. Faust , Nat. Chem. 2016, 8, 990–991. 27768096 10.1038/nchem.2656

[3] A. Corma , H. García , F. X. Llabrés i Xamena , Chem. Rev. 2010, 110, 4606–4655. 20359232 10.1021/cr9003924

[4] L. E. Kreno , K. Leong , O. K. Farha , M. Allendorf , R. P. Van Duyne , J. T. Hupp , Chem. Rev. 2012, 112, 1105–1125. 22070233 10.1021/cr200324t

[5] P. Horcajada , R. Gref , T. Baati , P. K. Allan , G. Maurin , P. Couvreur , G. Férey , R. E. Morris , C. Serre , Chem. Rev. 2012, 112, 1232–1268. 22168547 10.1021/cr200256v

[6a] H. Deng , C. J. Doonan , H. Furukawa , R. B. Ferreira , J. Towne , C. B. Knobler , B. Wang , O. M. Yaghi , Science 2010, 327, 846–850; 20150497 10.1126/science.1181761

[6b] M. Lammert , S. Bernt , F. Vermoortele , D. E. De Vos , N. Stock , Inorg. Chem. 2013, 52, 8521–8528. 23829498 10.1021/ic4005328

[7a] J. Jiang , O. M. Yaghi , Chem. Rev. 2015, 115, 6966–6997; 26088535 10.1021/acs.chemrev.5b00221

[7b] H. Furukawa , U. Müller , O. M. Yaghi , Angew. Chem. Int. Ed. 2015, 54, 3417–3430; 10.1002/anie.201410252 25586609

[7c] K. M. Choi , H. J. Jeon , J. K. Kang , O. M. Yaghi , J. Am. Chem. Soc. 2011, 133, 11920–11923; 21749096 10.1021/ja204818q

[7d] Z. Fang , B. Bueken , D. E. De Vos , R. A. Fischer , Angew. Chem. Int. Ed. 2015, 54, 7234–7254; 10.1002/anie.201411540 PMC4510710 26036179

[8] S. Marx , W. Kleist , A. Baiker , J. Catal. 2011, 281, 76–87.

[9a] N. Stock , S. Biswas , Chem. Rev. 2012, 112, 933–969; 22098087 10.1021/cr200304e

[9b] I. Agirrezabal-Telleria , I. Luz , M. A. Ortuño , M. Oregui-Bengoechea , I. Gandarias , N. López , M. A. Lail , M. Soukri , Nat. Commun. 2019, 10, 2076. 31061386 10.1038/s41467-019-10013-6 PMC6502813

[10] W. Zhang , M. Kauer , O. Halbherr , K. Epp , P. Guo , M. I. Gonzalez , D. J. Xiao , C. Wiktor , F. X. Liabrés i Xamena , C. Wöll , Y. Wang , M. Muhler , R. A. Fischer , Chem. Eur. J. 2016, 22, 14297–14307. 27529415 10.1002/chem.201602641

[11] H. Noei , O. Kozachuk , S. Amirjalayer , S. Bureekaew , M. Kauer , R. Schmid , B. Marler , M. Muhler , R. A. Fischer , Y. Wang , J. Phys. Chem. C 2013, 117, 5658–5666.

[12] Z. Fang , J. P. Dürholt , M. Kauer , W. Zhang , C. Lochenie , B. Jee , B. Albada , N. Metzler-Nolte , A. Pöppl , B. Weber , M. Muhler , Y. Wang , R. Schmid , R. A. Fischer , J. Am. Chem. Soc. 2014, 136, 9627–9636. 24915512 10.1021/ja503218j

[13] O. Kozachuk , I. Luz , F. X. Llabrés i Xamena , H. Noei , M. Kauer , H. B. Albada , E. D. Bloch , B. Marler , Y. Wang , M. Muhler , R. A. Fischer , Angew. Chem. Int. Ed. 2014, 53, 7058–7062; 10.1002/anie.201311128 24838592

[14] X. Kong , H. Deng , F. Yan , J. Kim , J. A. Swisher , B. Smit , O. M. Yaghi , J. A. Reimer , Science 2013, 341, 882–885. 23887875 10.1126/science.1238339

[15] A. M. Katzenmeyer , J. Canivet , G. Holland , D. Farrusseng , A. Centrone , Angew. Chem. Int. Ed. 2014, 53, 2852–2856; 10.1002/anie.201309295 24615798

[16] W. Schrimpf , J. Jiang , Z. Ji , P. Hirschle , D. C. Lamb , O. M. Yaghi , S. Wuttke , Nat. Commun. 2018, 9, 1647. 29695805 10.1038/s41467-018-04050-w PMC5916894

[17] L. Liu , Z. Chen , J. Wang , D. Zhang , Y. Zhu , S. Ling , K.-W. Huang , Y. Belmabkhout , K. Adil , Y. Zhang , B. Slater , M. Eddaoudi , Y. Han , Nat. Chem. 2019, 11, 622–628. 31086300 10.1038/s41557-019-0263-4

[18a] M. G. O′Brien , A. M. Beale , B. M. Weckhuysen , Chem. Soc. Rev. 2010, 39, 4767–4782; 20978667 10.1039/c0cs00088d

[18b] B. M. Weckhuysen , Chem. Soc. Rev. 2010, 39, 4557–4559.

[19] J. P. Dürholt , J. Keupp , Schmid , Rochus , Eur. J. Inorg. Chem. 2016, 4517–4523.

[20] C. Prestipino , L. Regli , J. G. Vitillo , F. Bonino , A. Damin , C. Lamberti , A. Zecchina , P. L. Solari , K. O. Kongshaug , S. Bordiga , Chem. Mater. 2006, 18, 1337–1346.

[21a] N. J. Everall , Analyst 2010, 135, 2512–2522; 20725670 10.1039/c0an00371a

[21b] N. J. Everall , Appl. Spectrosc. 2009, 63, 245A–262A; 10.1366/000370209789379196 19796478

[21c] N. J. Everall , Appl. Spectrosc. 2000, 54, 773–782.

[22] C.-S. Tsao , M.-S. Yu , T.-Y. Chung , H.-C. Wu , C.-Y. Wang , K.-S. Chang , H.-L. Chen , J. Am. Chem. Soc. 2007, 129, 15997–16004. 18044895 10.1021/ja0752336

[23] A. Guinier , G. Fournet , Small angle scattering of X-rays, Wiley, New York, 1955.

[24] S. Lowell , J. E. Shields , M. A. Thomas , M. Thommes in Characterization of Porous Solids and Powders: Surface Area, Pore Size and Density, Springer, Dordrecht, 2004, pp. 15–57.

[25] A. Umemura , S. Diring , S. Furukawa , H. Uehara , T. Tsuruoka , S. Kitagawa , J. Am. Chem. Soc. 2011, 133, 15506–15513. 21861521 10.1021/ja204233q

[26] M. J. Van Vleet , T. Weng , X. Li , J. R. Schmidt , Chem. Rev. 2018, 118, 3681–3721. 29514005 10.1021/acs.chemrev.7b00582

[27a] Q. Liu , J.-M. Yang , L.-N. Jin , W.-Y. Sun , CrystEngComm 2016, 18, 4127–4132;

[27b] Q. Liu , J.-M. Yang , L.-N. Jin , W.-Y. Sun , Chem. Eur. J. 2014, 20, 14783–14789; 25224902 10.1002/chem.201402923

[27c] Y. C. Tan , H. C. Zeng , Adv. Funct. Mater. 2017, 27, 1703765.

[28a] Q. Liu , L.-N. Jin , W.-Y. Sun , Chem. Commun. 2012, 48, 8814–8816; 10.1039/c2cc34192a 22836446

[28b] S. Diring , S. Furukawa , Y. Takashima , T. Tsuruoka , S. Kitagawa , Chem. Mater. 2010, 22, 4531–4538.

[29] F. Wang , H. Guo , Y. Chai , Y. Li , C. Liu , Microporous Mesoporous Mater. 2013, 173, 181–188.

[30] M. R. Armstrong , S. Senthilnathan , C. J. Balzer , B. Shan , L. Chen , B. Mu , Ultra. Sonochem. 2017, 34, 365–370. 10.1016/j.ultsonch.2016.06.011 27773257

[31] C. Xu , C. Wang , T. Zheng , Q. Hu , C. Bai , CrystEngComm 2018, 20, 7275–7280.

[32] J. Zhang , H. Li , Q. Kuang , Z. Xie , Acc. Chem. Res. 2018, 51, 2880–2887. 30346701 10.1021/acs.accounts.8b00344

[33] M. P. Attfield , P. Cubillas , Dalton Trans. 2012, 41, 3869–3878. 22183082 10.1039/c2dt12006b

[34] J. D. Hancock , J. H. Sharp , J. Am. Ceram. Soc. 1972, 55, 74–77.

[35a] D. Zacher , J. Liu , K. Huber , R. A. Fischer , Chem. Commun. 2009, 1031–1033; 10.1039/b819580c 19225626

[35b] F. Millange , R. El Osta , M. E. Medina , R. I. Walton , CrystEngComm 2011, 13, 103–108.

[36] V. K. LaMer , R. H. Dinegar , J. Am. Chem. Soc. 1950, 72, 4847–4854.

[37] X.-G. Wang , Q. Cheng , Y. Yu , X.-Z. Zhang , Angew. Chem. Int. Ed. 2018, 57, 7836–7840; 10.1002/anie.201803766 29700914

[38] J.-C. Lee , J.-O. Kim , H.-J. Lee , B. Shin , S. Park , Chem. Mater. 2019, 31, 7377–7385.

[39] G. Mahalakshmi , V. Balachandran , Spectrochim. Acta Part A 2014, 124, 535–547. 10.1016/j.saa.2014.01.061 24508892

[40a] J. Szanyi , M. Daturi , G. Clet , D. R. Baer , C. H. F. Peden , Phys. Chem. Chem. Phys. 2012, 14, 4383–4390; 22354204 10.1039/c2cp23708c

[40b] L. Alaerts , E. Séguin , H. Poelman , F. Thibault-Starzyk , P. A. Jacobs , D. E. de Vos , Chem. Eur. J. 2006, 12, 7353–7363; 16881030 10.1002/chem.200600220

[40c] M. Todaro , L. Sciortino , F. M. Gelardi , G. Buscarino , J. Phys. Chem. C 2017, 121, 24853–24860;

[40d] V. L. Sushkevich , A. V. Smirnov , J. A. van Bokhoven , J. Phys. Chem. C 2019, 123, 9926–9934;

[40e] V. L. Sushkevich , J. A. van Bokhoven , Chem. Commun. 2018, 54, 7447–7450. 10.1039/c8cc03921f 29911214

[41a] B. Jee , M. Hartmann , A. Pöppl , Mol. Phys. 2013, 111, 2950–2966;

[41b] F. Gul-E-Noor , M. Mendt , D. Michel , A. Pöppl , H. Krautscheid , J. Haase , M. Bertmer , J. Phys. Chem. C 2013, 117, 7703–7712; 10.1063/1.4813613 23883020

[41c] A. Pöppl , S. Kunz , D. Himsl , M. Hartmann , J. Phys. Chem. C 2008, 112, 2678–2684.

[42] M. Todaro , A. Alessi , L. Sciortino , S. Agnello , M. Cannas , F. M. Gelardi , G. Buscarino , J. Spectr. 2016, 2016, 54 8074297.

[43] K. N. Wong , S. D. Colson , J. Mol. Spectrosc. 1984, 104, 129–151.

[44] D.-Y. Wu , B. Ren , Y.-X. Jiang , X. Xu , Z.-Q. Tian , J. Phys. Chem. A 2002, 106, 9042–9052.

[45] E. Pérez-Mayoral , Z. Musilová , B. Gil , B. Marszalek , M. Položij , P. Nachtigall , J. Čejka , Dalton Trans. 2012, 41, 4036–4044. 22293862 10.1039/c2dt11978a

[46a] C. Volkringer , H. Leclerc , J.-C. Lavalley , T. Loiseau , G. Férey , M. Daturi , A. Vimont , J. Phys. Chem. C 2012, 116, 5710–5719;

[46b] A. Dhakshinamoorthy , M. Alvaro , P. Horcajada , E. Gibson , M. Vishnuvarthan , A. Vimont , J.-M. Grenèche , C. Serre , M. Daturi , H. Garcia , ACS Catal. 2012, 2, 2060–2065;

[46c] H. Leclerc , A. Vimont , J.-C. Lavalley , M. Daturi , A. D. Wiersum , P. L. Llwellyn , P. Horcajada , G. Ferey , C. Serre , Phys. Chem. Chem. Phys. 2011, 13, 11748–11756. 21597609 10.1039/c1cp20502a

[47] M. S. Holm , S. Svelle , F. Joensen , P. Beato , C. H. Christensen , S. Bordiga , M. Bjørgen , Appl. Catal. A 2009, 356, 23–30.

[48] J. H. Kwak , J. H. Lee , S. D. Burton , A. S. Lipton , C. H. F. Peden , J. Szanyi , Angew. Chem. Int. Ed. 2013, 52, 9985–9989; 10.1002/anie.201303498 23939905

[49] K. I. Hadjiivanov , Catal. Rev. 2000, 42, 71–144.

[50] K. I. Hadjiivanov , G. N. Vayssilov , Adv. Catal. 2002, 47, 307–511.

[51] P. St. Petkov , G. N. Vayssilov , J. Liu , O. Shekhah , Y. Wang , C. Wöll , T. Heine , ChemPhysChem 2012, 13, 2025–2029. 22517762 10.1002/cphc.201200222

[52] N. Drenchev , E. Ivanova , M. Mihaylov , K. Hadjiivanov , Phys. Chem. Chem. Phys. 2010, 12, 6423–6427. 20393653 10.1039/c000949k

[53] Y. Liu , F. Meirer , P. A. Williams , J. Wang , J. C. Andrews , P. Pianetta , J. Synchrotron Radiat. 2012, 19, 281–287. 22338691 10.1107/S0909049511049144 PMC3284347

[54] V. Zdravkova , N. Drenchev , E. Ivanova , M. Mihaylov , K. Hadjiivanov , J. Phys. Chem. C 2015, 119, 15292–15302.

[55] C. E. Ruggiero , S. M. Carrie , W. E. Antholine , J. W. Whittaker , C. J. Cramer , W. B. Tolman , J. Am. Chem. Soc. 1993, 115, 11285–11298.

[56] M. Rivera-Torrente , M. Filez , C. Schneider , E. C. van der Feltz , K. Wolkersdörfer , D. H. Taffa , M. Wark , R. A. Fischer , B. M. Weckhuysen , Phys. Chem. Chem. Phys. 2019, 21, 25678–25689. 31742269 10.1039/c9cp05082e

