# Supplementary material for: Multi‐Spectroscopic Interrogation of the Spatial Linker Distribution in Defect‐Engineered Metal–Organic Framework Crystals: The [Cu3(btc)2−x(cydc)x] Showcase
Source: Chemistry. 2020 Mar 3;26(16):3614–25. doi: 10.1002/chem.201905645 (PMC7154733; doi:10.1002/chem.201905645)
Supplement: Supplementary file 1 — Supplementary [file CHEM-26-3614-s001.pdf]

CHEMISTRY
A European Journal

Supporting Information

Multi-Spectroscopic Interrogation of the Spatial Linker Distribution in Defect-Engineered Metal–Organic Framework Crystals: The $[Cu_3(btc)_{2-x}(cydc)_x]$ Showcase**

Miguel Rivera-Torrente, Matthias Filez, Florian Meirer, and Bert M. Weckhuysen*[a]

chem_201905645_sm_miscellaneous_information.pdf

Table of Contents

1. **Materials and Methods**
2. **High-Performance Liquid Chromatography Analysis**
3. **Raman Micro-Spectroscopy of Pure Linker Compounds and Maps of $[Cu_3(BTC)_{1.4}(CYDC)_{0.6}]$ Crystals**
4. **Evolution of Parameters with CYDC Concentration Calculated from X-ray Diffraction**
5. **Nitrogen Adsorption Isotherms at 77 K**
6. **Calculated Microspecies Distribution with pH of BTC and CYDC**
7. **Diffuse Reflectance UV-vis Spectroscopy of the Pure Linker Compound**
8. **Electron Paramagnetic Resonance**

1. MATERIALS AND METHODS

General information: N,N-dimethylformamide (DMF, $HCON(CH_3)_2$, ≥99.8%), copper nitrate trihydrate ($Cu(NO_3)_2{\cdot}3H_2O$, 99%), trimesic acid (H_3BTC, $C_9H_6O_6$, 99%), 5-cyano-1,3-benzenedicarboxylic acid (CYDC, $C_9H_5NO_4$, ≥95%) were all purchased from Sigma-Aldrich. Ethanol (technical grade, 97%), dichloromethane (technical grade, 99%) and acetone (≥99.5%) were purchased from Biosolve^{TM} or VWR International.

Synthesis of [$Cu_3(BTC)_{2-x}(CYDC)_x$]: The crystals were synthesized according to the protocol described in the literature.[1] Briefly, $Cu(NO_3)_2{\cdot}3H_2O$ (0.398 g, 1.61 mmol) were dissolved into 6 mL of deionized (DI) water. Then, *L-x* moles of H_3BTC and *x* moles of CYDC (see Table S1) were dissolved into a mixture of N,N-DMF (6 mL) and EtOH (6 mL) by ultrasonication at room temperature for 5 min. Thereafter, the metal and linker solutions were mixed in a sealed glass 25 mL scintillation vial and heated to 343 K in an oven for 12 h. After cooling down to room temperature naturally, the powders were collected by filtration and washed with ethanol (20 mL), acetone (20 mL) and dichloromethane (20 mL), and dried in air at room temperature.

Table S1. Mass compositions of the initial mixtures for $Cu_3(BTC)_{2-x}(CYDC)_x$.

CYDC mol (%)*	$Cu(NO_3)_2{\cdot}3H_2O$ (g)	H_3BTC (g)	CYDC (g)
**Parent (0%)**	0.389	0.226	-
**10%**	0.389	0.203	0.020
**30%**	0.389	0.158	0.062
**50%**	0.389	0.113	0.103
**70%**	0.389	0.068	0.144

*Moles percentage of CYDC in the starting synthesis mixture. **Mole percentage of CYDC incorporated into the lattice as determined by HPLC analysis.

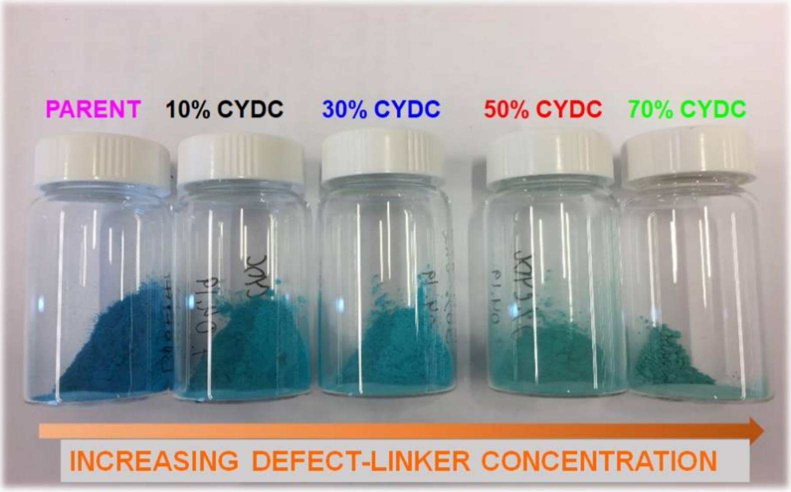

Figure S1. Different defect-engineered [$Cu_3(BTC)_{2-x}(CYDC)_x$] materials showing the concentration of CYDC in the starting synthesis mixture.

2. High-Performance Liquid Chromatography Analysis

High-Performance Liquid Chromatography (HPLC) analyses were done in order to evaluate the incorporation of CYDC linker into the framework after crystallization and washing. Briefly, about ~ 20 mg of evacuated MOF were dissolved in 10%TFA/DMSO (trifluoroacetic acid, Biosolve^{TM}, HPLC grade; dimethylsulfoxide, Sigma Aldrich, 99.9% anhydrous) v/v under sonication for 10 min, and filtered with PTFE (0.45 µm) filters. The water used as an eluent was Milli-Q water (18.2 $S{\cdot}cm^{-1}$ at 298 K) and the acetonitrile was HPLC grade (Sigma). Then, the samples were separated under the following conditions:

A	water/MeCN/TFA – 95/5/0.1 (%, v/v/v)
B	MeCN/water/TFA – 95/5/0.1 (%, v/v/v)

Gradient: start with 100% A for 5 min; then in 15 min to 75% B (i.e. 5%/min); steady at 75% B for 2.5 min; drop to 100% A in 5 min (i.e. 20%/min); steady at 100% A for 2.5 min. ***Column:*** SunFire C18 Column, 100Å, 3.5 µm, 4.6 mm X 150 mm. ***Detection:*** λ = 220 nm and λ = 254 nm, for the calibration curves the absorption at 254 nm was used. Waters R2451 detector. ***Equipment:*** HPLC-runs were performed on a Waters HPLC instrument.

Calibration curves with 10 different concentrations for each component, *i.e.* 1,3,5-benzenetricarboxylic acid and 5-cyano-1,3-dicarboxylic acid in 10% trifluoroacetic acid/DMSO in the range:

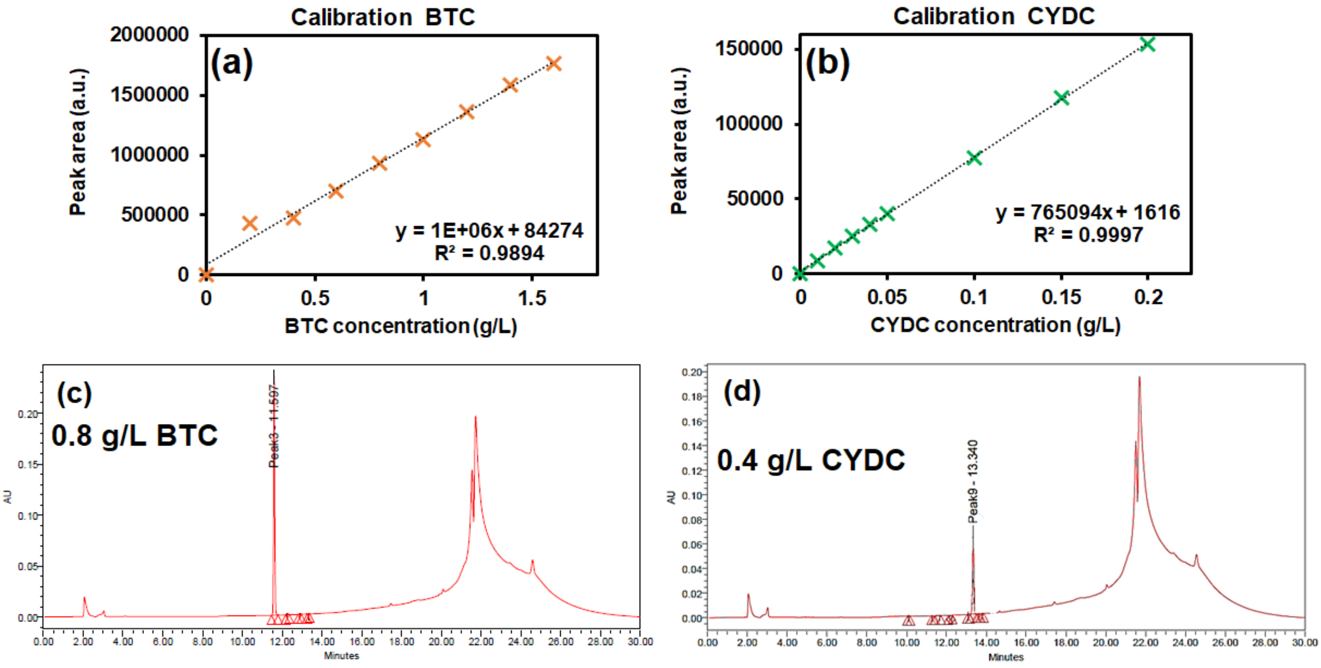

Figure S2. Calibration curve of different concentrations of the peaks at (a) $t_R \approx 11.6$ min for BTC and (b) $t_R \approx 13.4$ min for CYDC of the chromatographic system used in the quantification. Note that the concentration range for the defect linker is lower than in the case of BTC, but the response factor is linear. Exemplary chromatograms of the pure components injected showing retention times (c) BTC and (d) CYDC.

As described in previous literature, quantification of the incorporated CYDC linker into the lattice is challenging, given that accurate solid-state NMR is hindered by the paramagnetic Cu^{2+} cations in the lattice and the method used for dissolving the lattice results into decomposition of the linker into multiple products. This was also observed in our case, where the peak corresponding to CYDC in the samples after digestion split into different eluted compounds (Figure S3). We hypothesize that the decomposition to products *via* hydrolysis of the nitrile group to the amide, subsequent formation of ammonium salts or oxidation is likely under the digestion conditions (TFA/DMSO and Cu^{2+} ions present).[2]

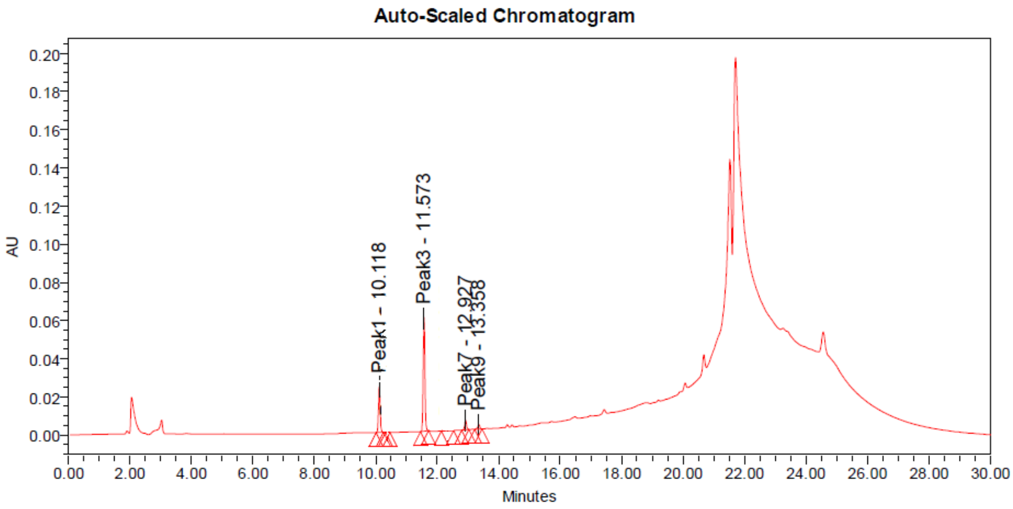

Figure S3. Chromatogram of the sample prepared with 70 %mol of CYDC in the mixture. The peak at t_R = 13.6 min shows very low intensity at the expense of new peaks at t_R = 10.2 min and t_R = 12.9 min not observed when injecting the pure compounds.

3. Evolution of Parameters with CYDC Concentration Calculated from X-ray Diffraction

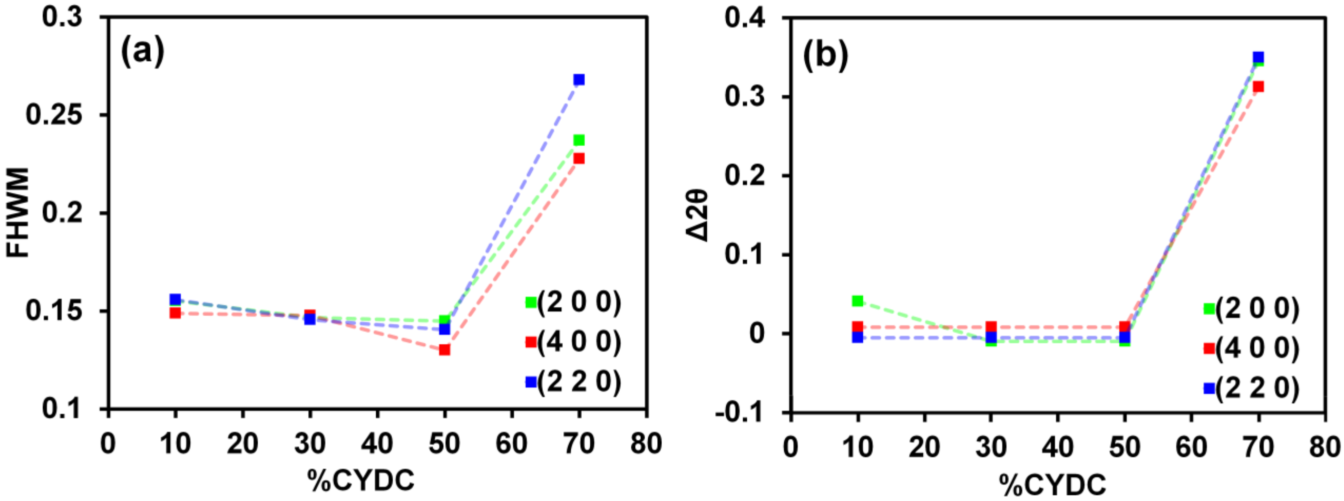

Figure S4. (a) Evolution of the full width at half maximum (FWHM) values with increasing molar concentration of the defective linker in the lattice. (b) Shift of the peaks corresponding to the (200), (400) and (220) reflections.

Table S2. Lattice parameters of the CYDC-containing HKUST-1 crystals with composition [$Cu_3(BTC)_{2-x}(CYDC)_x$] obtained from the Bragg equation for different reflections.

h k l	**2θ (°)**	***d* (Å)**			
		x = 0.2	x = 0.6	x = 1.0	x = 1.4
(2 0 0)	7.8	13.15	13.15	13.15	12.60
(2 2 0)	11.1	9.30	9.30	9.30	9.02
(2 2 2)	13.5	7.59	7.59	7.59	7.41

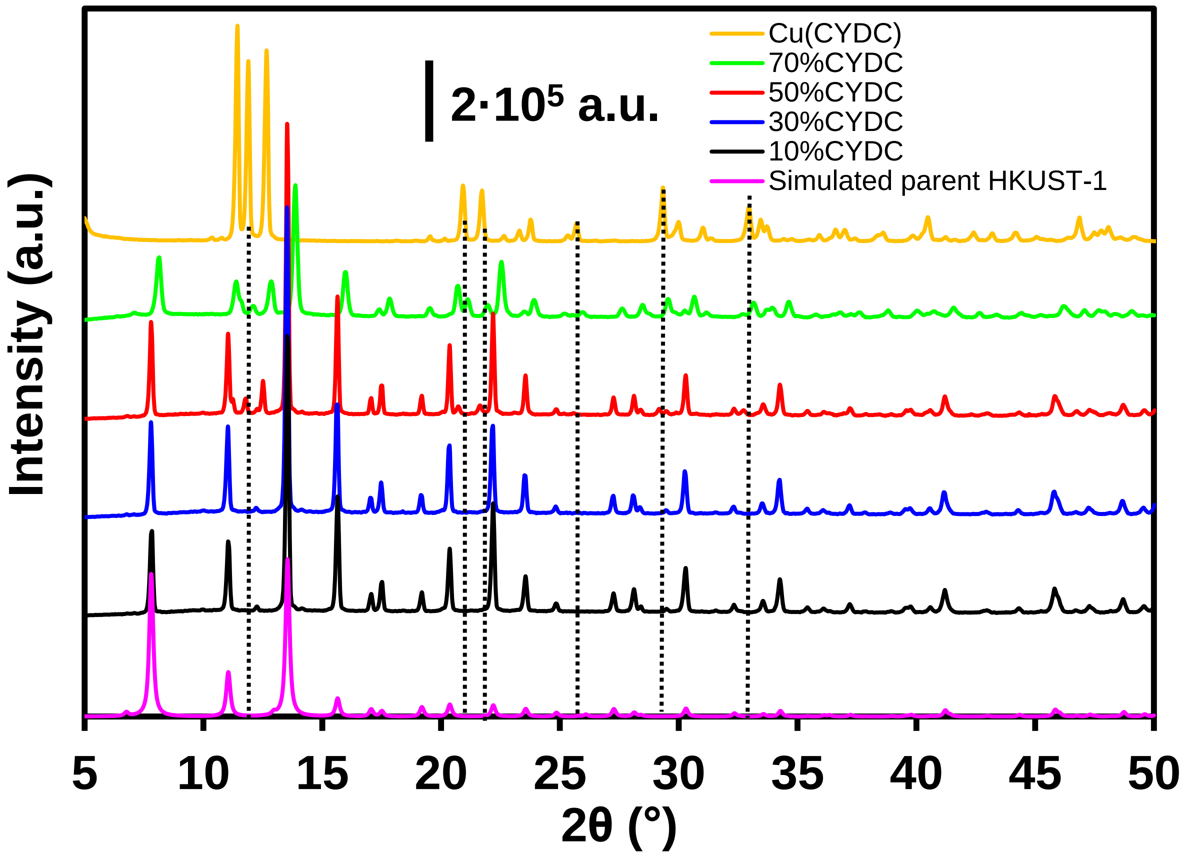

Figure S5. Comparison of the XRD patterns collected for the $[Cu_3(BTC)_{2-x}(CYDC)_x]$ materials with increasing amount of CYDC in the framework to the crystalline powder by using only CYDC as a linker under the same synthesis conditions.

4. Nitrogen Adsorption Isotherms at 77 K

Determination of the Brunauer-Emmett-Teller (BET) specific surface areas was carried out following the consistency criteria proposed by Lowell *et al.*[3], Rouquerol *et al.*,[4] Walton *et al*.,[5] De Lange *et al.*[6] and Gómez-Gualdrón *et al.*[7] Linear fitting of the values through the BET approach was done in the 0.015 ≤ p/p_0 ≤ 0.021 range of the isotherms (with 6 points in the linear plot, *i.e.* enough degrees of freedom) and maintaining a positive C-value. *Note that some of the C values are C >> 100, indicating strong adsorption onto the surfaces and interactions with the pores.

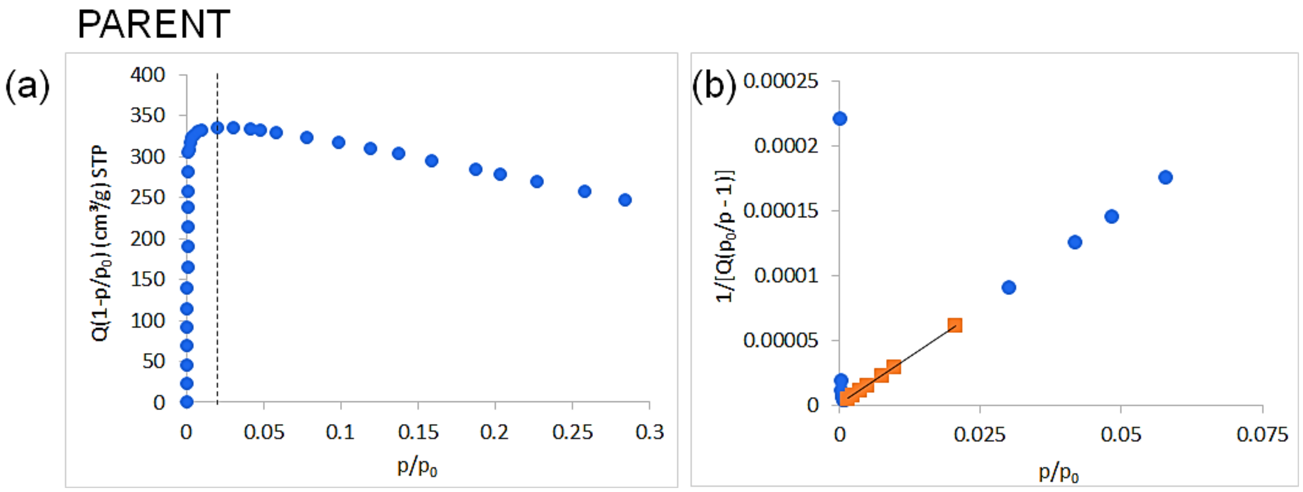

**BET surface area:**	1470 ± 1 m²/g
**Slope:**	0.002960 ± 0.000001 g/cm³ STP
**Y-intercept:**	0.000000 ± 0.000000 g/cm³ STP
**C:**	6171.5
**Q_m:**	337.79 cm³/g STP
**Correlation coefficient:**	0.9999995
**Molecular cross-sectional area:**	0.1620 nm²

Figure S6. (a) *Q(1-p/p₀) vs*. Relative pressure and (b) linear BET fit plots obtained from the nitrogen physisorption isotherms at 77 K of the parent HKUST-1 material. The dashed black line shows the maximum increase value at p/p_0 = 0.025; and thus, this was the maximum value used for determining the BET area (orange data points) in (b), *i.e.* enough degrees of freedom. The table shows the values of the different relevant parameters, including C > 0 and R^2 > 0.999.

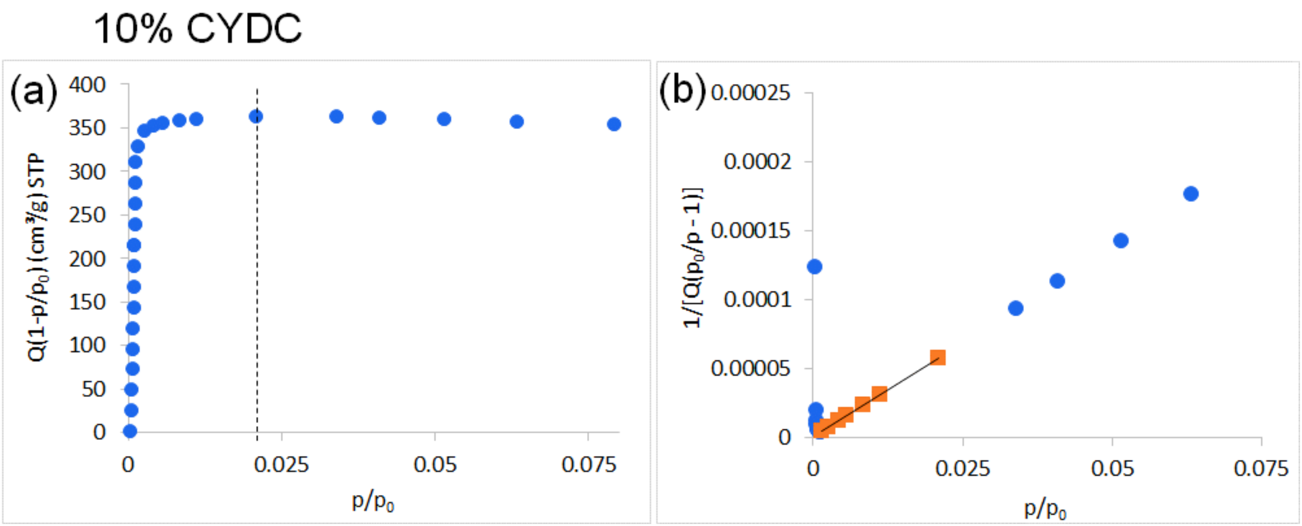

**BET surface area:**	1592 ± 1 m²/g
**Slope:**	0.002732 ± 0.000001 g/cm³ STP
**Y-intercept:**	0.000000 ± 0.000000 g/cm³ STP
**C:**	6301.87
**Q_m:**	365.92 cm³/g STP
**Correlation coefficient:**	0.9999993
**Molecular cross-sectional area:**	0.1620 nm²

Figure S7. (a) *Q(1-p/p₀) vs*. Relative pressure and (b) linear BET fit plots obtained from the nitrogen physisorption isotherms at 77 K of the $[Cu_3(BTC)_{1.8}(CYDC)_{0.2}]$ material. The dashed black line shows the maximum increase value at p/p_0 = 0.025; and thus, this was the maximum value used for determining the BET area (orange data points) in (b), *i.e.* enough degrees of freedom. The table shows the values of the different relevant parameters, including C > 0 and R^2 > 0.999.

30% CYDC

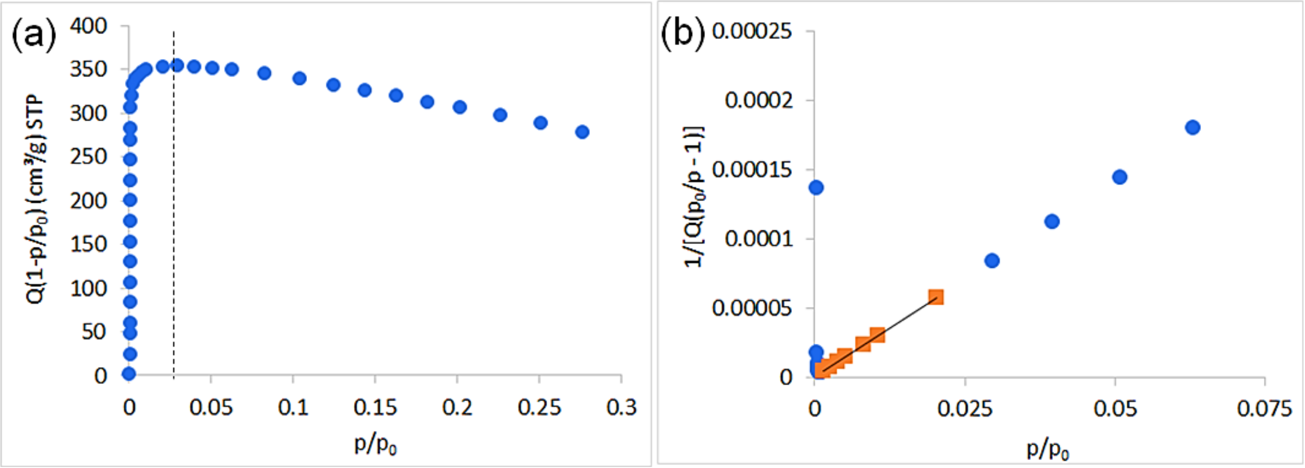

**BET surface area:**	1550 ± 2 m²/g
**Slope:**	0.002807 ± 0.000004 g/cm³ STP
**Y-intercept:**	0.000001 ± 0.000000 g/cm³ STP
**C:**	5377.60
**Q_m:**	356.22 cm³/g STP
**Correlation coefficient:**	0.9999954
**Molecular cross-sectional area:**	0.1620 nm²

Figure S8. (a) *Q(1-p/p₀) vs*. Relative pressure and (b) linear BET fit plots obtained from the nitrogen physisorption isotherms at 77 K of the $[Cu_3(BTC)_{1.4}(CYDC)_{0.6}]$ material. The dashed black line shows the maximum increase value at p/p_0 = 0.025; and thus, this was the maximum value used for determining the BET area (orange data points) in (b), *i.e.* enough degrees of freedom. The table shows the values of the different relevant parameters, including C > 0 and R^2 > 0.999.

50% CYDC

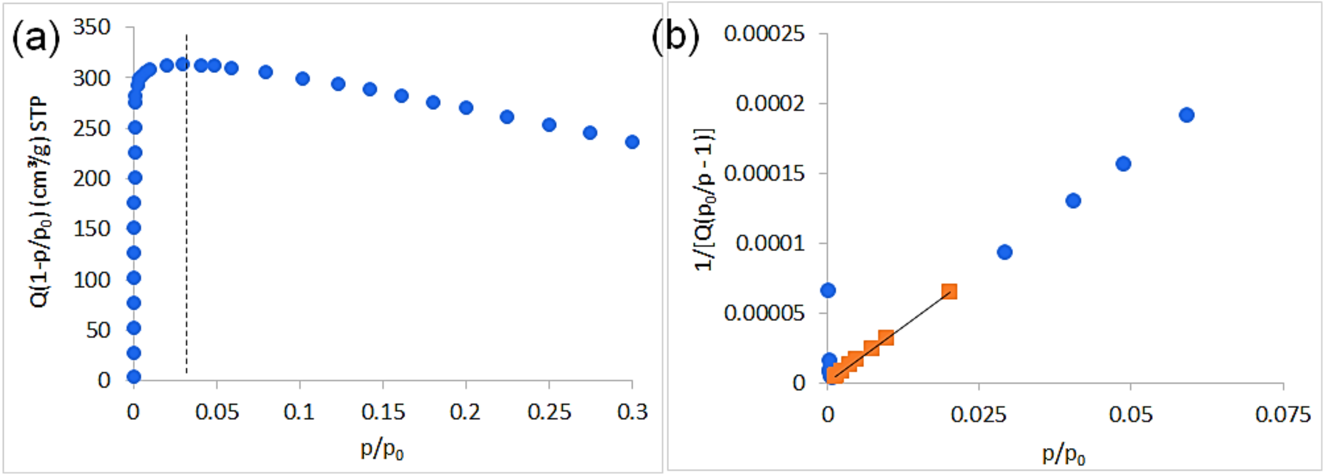

**BET surface area:**	1372 ± 2 m²/g
**Slope:**	0.003170 ± 0.000004 g/cm³ STP
**Y-intercept:**	0.000001 ± 0.000000 g/cm³ STP
**C:**	5052.11
**Q_m:**	315.36 cm³/g STP
**Correlation coefficient:**	0.9999948
**Molecular cross-sectional area:**	0.1620 nm²

Figure S9. (a) *Q(1-p/p₀) vs*. Relative pressure and (b) linear BET fit plots obtained from the nitrogen physisorption isotherms at 77 K of the $[Cu_3(BTC)_{1.0}(CYDC)_{1.0}]$ material. The dashed black line shows the maximum increase value at p/p_0 = 0.025; and thus, this was the maximum value used for determining the BET area (orange data points) in (b), *i.e.* enough degrees of freedom. The table shows the values of the different relevant parameters, including C > 0 and R^2 > 0.999.

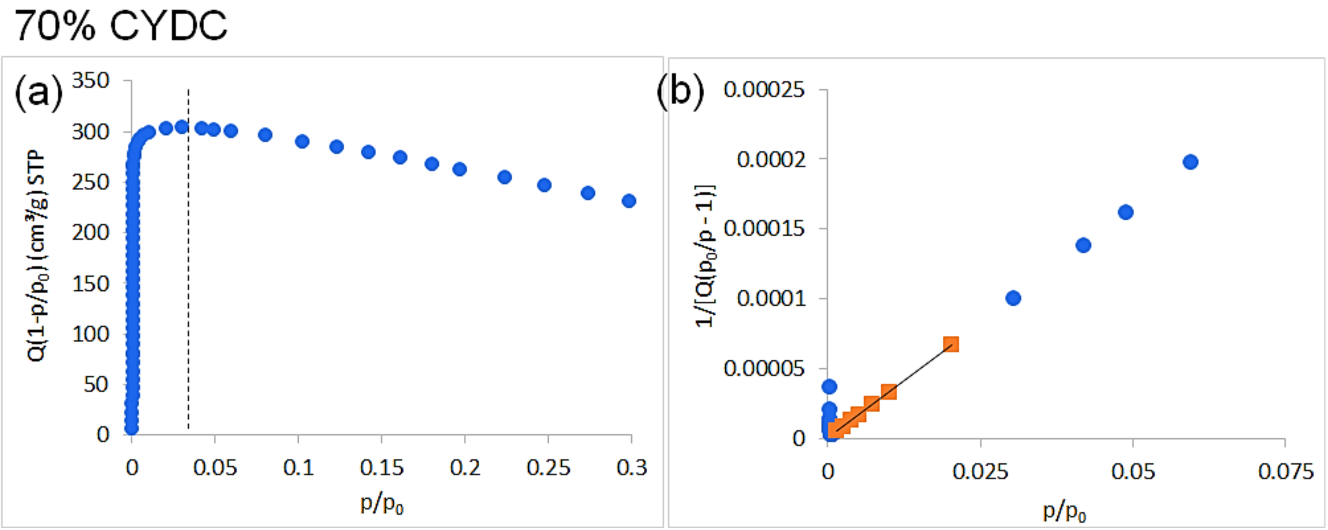

**BET surface area:**	1331 ± 3 m²/g
**Slope:**	0.003270 ± 0.000007 g/cm³ STP
**Y-intercept:**	0.000001 ± 0.000000 g/cm³ STP
**C:**	5146.24
**Q_m:**	305.76 cm³/g STP
**Correlation coefficient:**	0.9999890
**Molecular cross-sectional area:**	0.1620 nm²

Figure S10. (a) *Q(1-p/p₀) vs*. Relative pressure and (b) linear BET fit plots obtained from the nitrogen physisorption isotherms at 77 K of the $[Cu_3(BTC)_{0.6}(CYDC)_{1.4}]$ material. The dashed black line shows the maximum increase value at p/p_0 = 0.025; and thus, this was the maximum value used for determining the BET area (orange data points) in (b), *i.e.* enough degrees of freedom. The table shows the values of the different relevant parameters, including C > 0 and R^2 > 0.999.

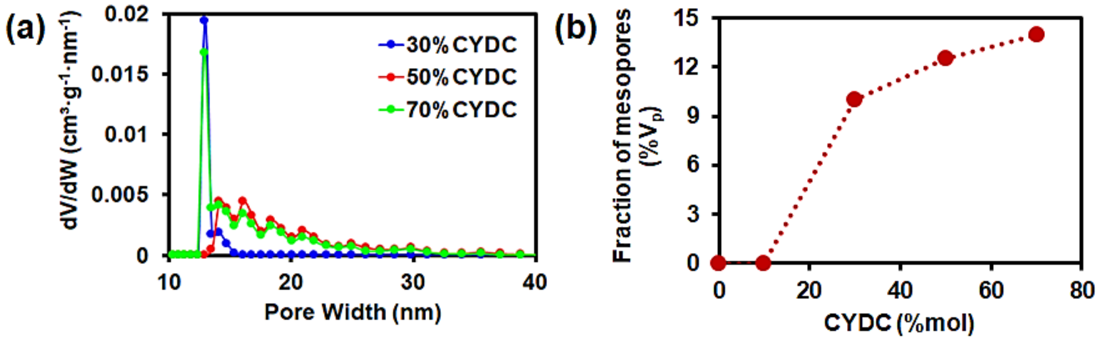

Figure S11. (a) Non-Local Density Functional Theory (NLDFT) pore size distribution at 77 K with slit pore geometry obtained from the nitrogen adsorption isotherms of the defect-engineered MOFs with 30 (blue), 50 (red) and 70 (green) %mol CYDC in the mixture. Only the *p/p_0* > 0.3 region was fitted with NLDFT for calculating

the mesopores. *NLDFT with oxidic surfaces and slit pores model has been utilized to carry out the fitting procedure. (b) Evolution of the mesopore fraction of the total pore volume with CYDC concentration in the lattice.

5. Calculated Microspecies Distribution with pH of BTC and CYDC

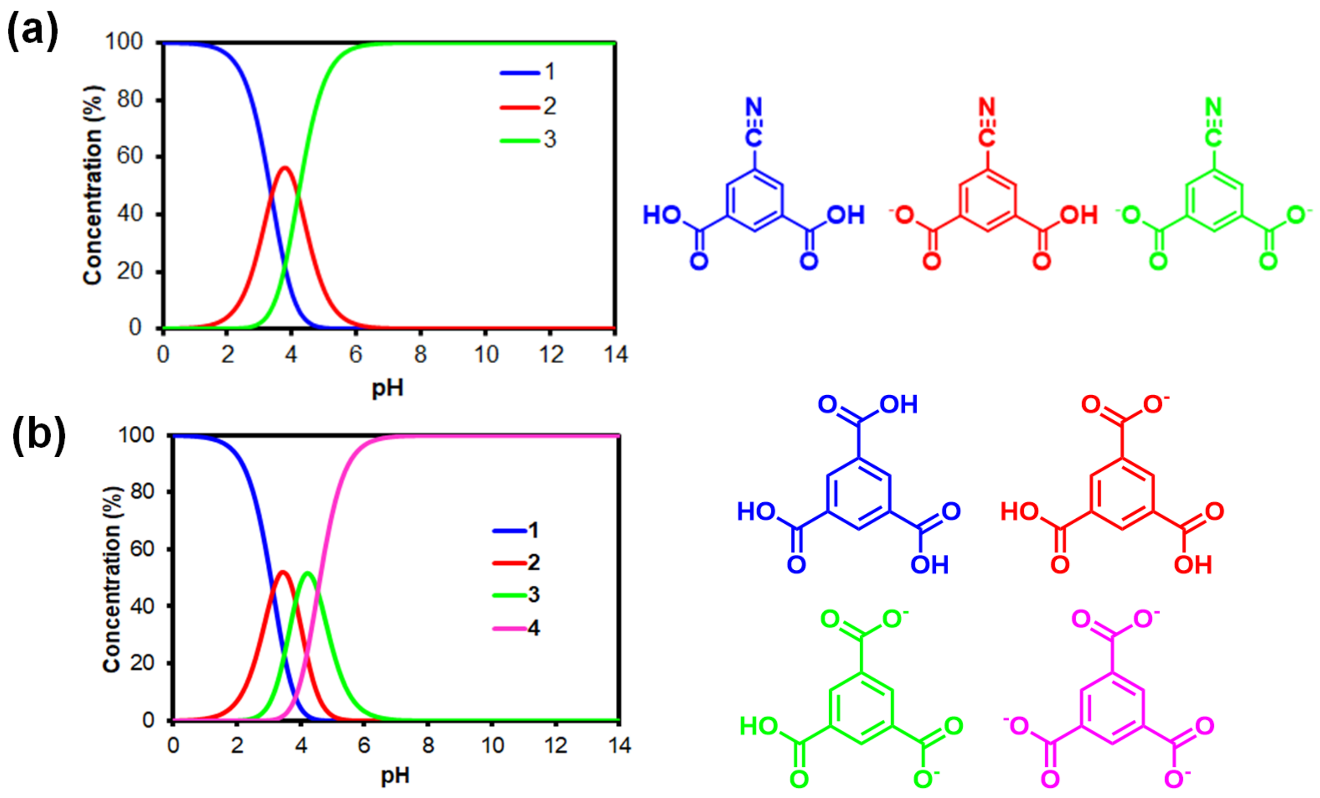

Figure S12. Calculated distribution of the different microspecies in aqueous solution at 298 K, 1 bar and ionic strength I = 0 of (a) 5-cyano-1,3-benzenedicarboxylic acid and (b) 1,3,5-benzenetricarboxylic acid. The distribution was obtained with the Reactor 19.2 package (MarvinView © ChemAxon).

6. Crystal Size Distributions of the Defect-Engineered [$Cu_3(BTC)_{2-x}(CYDC)_x$]

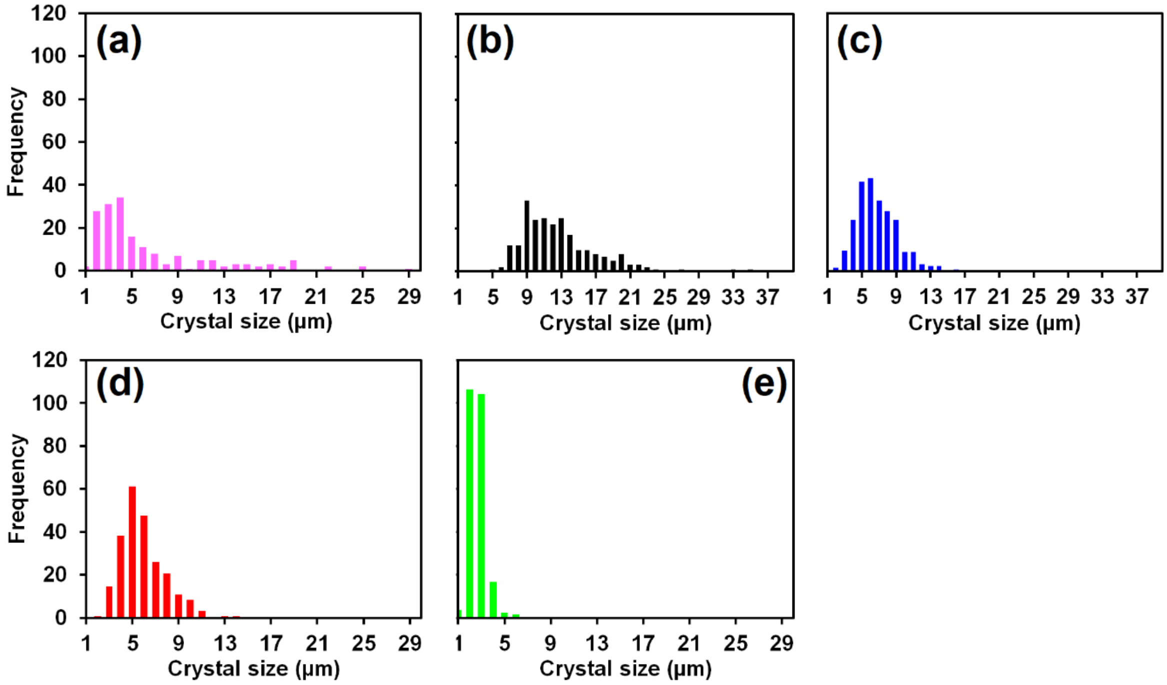

Figure S13. Crystal size distributions obtained from the scanning electron microscopy (SEM) images of the [$Cu_3(BTC)_{2-x}(CYDC)_x$] crystals prepared with following compositions: (a) [$Cu_3(BTC)_2$], (b) [$Cu_3(BTC)_{1.8}(CYDC)_{0.2}$], (c) [$Cu_3(BTC)_{1.6}(CYDC)_{0.4}$], (d) [$Cu_3(BTC)_{1.0}(CYDC)_{1.0}$] and (e) [$Cu_3(BTC)_{0.6}(CYDC)_{1.4}$].

7. Raman Micro-Spectroscopy of Pure Linker Compounds and [$Cu_3(BTC)_{1.4}(CYDC)_{0.6}$] Crystals

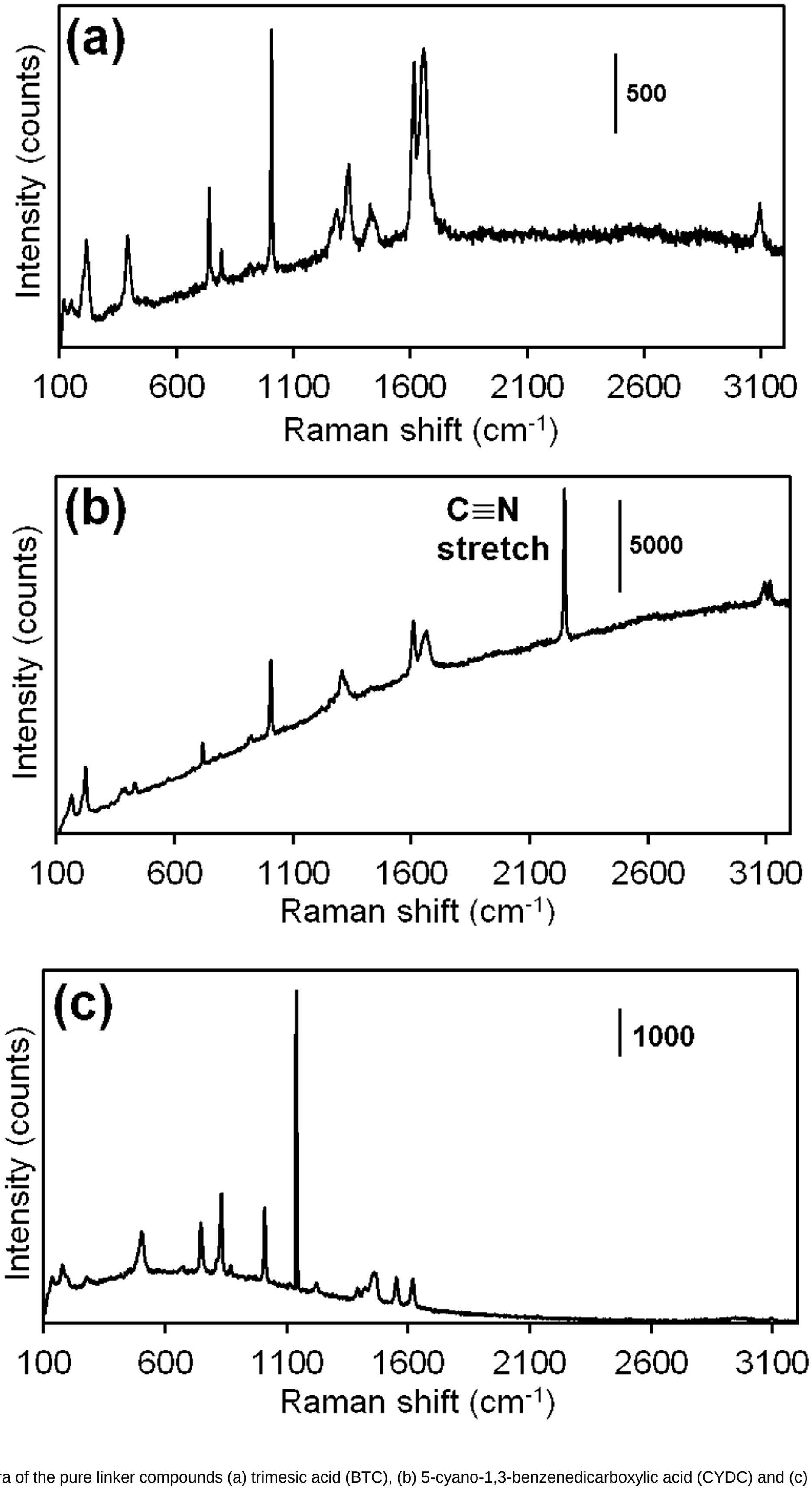

Figure S14. Raman spectra of the pure linker compounds (a) trimesic acid (BTC), (b) 5-cyano-1,3-benzenedicarboxylic acid (CYDC) and (c) parent Cu_3BTC_2. Note the peak at 2247 cm^{-1} corresponding to the C≡N stretch of the CYDC linker in spectrum (b).

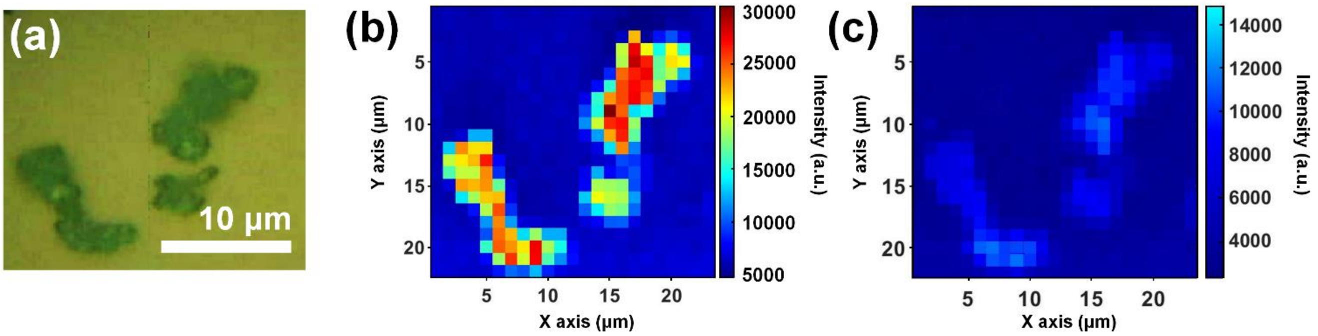

Figure S15. (a) Optical image of [$Cu_3(BTC)_{1.4}(CYDC)_{0.6}$] crystals with the corresponding Raman maps of the (b) 480-520 cm^{-1} and the (c) 2220-2260 cm^{-1} spectral regions. (d) Raman spectra of selected, exemplary pixels in (b) and (c) showing different contents of CYDC linker and Cu_3BTC_2 material. Note that the different areas show low intensity $\nu_{(C≡N)}$ stretching mode at 2240 cm^{-1} corresponding to heterogeneous distribution on the defect linker. Intensity of the maps corresponds to normalized signal-to-baseline. Note that the color intensity bar has been adjusted so that similar color shades match for both maps.

8. Diffuse Reflectance UV-vis Spectroscopy of the Pure Linker Compound

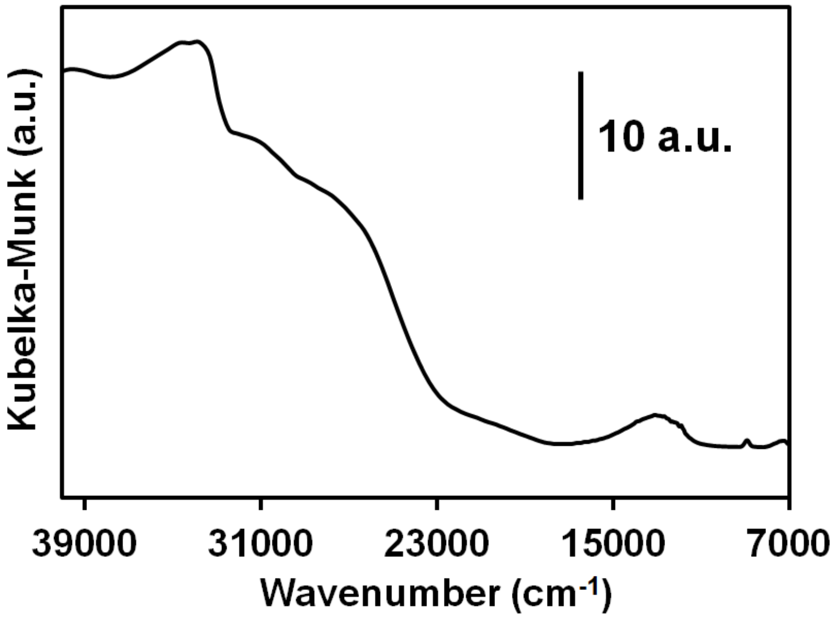

Figure S16. Diffuse reflectance UV-vis spectroscopy of pure 5-cyano-1,3-benzenedicarboxylic acid (CYDC). *Peak corresponding to the monochromator switch at 860 nm. Arrows in (a) indicate the presence of two distinct types of *d-d* transitions of Cu^{2+} ions in the lattice.

9. Electron Paramagnetic Resonance Spectroscopy

Table S3. Calculated parameters from the EPR spectra of the Cu^{2+} (S = 1/2, I = 3/2) ions in the frameworks with different CYDC contents.

CYDC (% mol)	B_0 (G)	ΔB_{pp} (G)	g factor (at B_0)	$g_{xx} = g_{yy}$	g_z	A_{zz} (MHz)
**Parent (0)**	3266.8	58.6	2.04436	2..04	2.37	-
**10**	3260.9	105.6	2.03711	2.06	2.35	$5.93 \cdot 10^{-3}$
**30**	3255.1	146.7	2.04355	2.07	2.36	$9.53 \cdot 10^{-3}$
**50**	3243.4	240.5	2.04450	2.06	2.34	$6.42 \cdot 10^{-4}$
**70**	3259.2	164.6	2.04515	2.06	2.37	$5.16 \cdot 10^{-3}$

References

[1] Z. Fang, J. P. Dürholt, M. Kauer, W. Zhang, C. Lochenie, B. Jee, B. Albada, N. Metzler-Nolte, A. Pöppl, B. Weber, M. Muhler, Y. Wang, R. Schmid, R. A. Fischer, *J. Am. Chem. Soc.* **2014**, *136*, 9627-9636.

[2] K. Omura, D. Swern, *Tetrahedron* **1978**, *34*, 1651-1660.

[3] S. Lowell, J. E. Shields, M. A. Thomas, M. Thommes, in *Characterization of Porous Solids and Powders: Surface Area, Pore Size and Density* (Eds.: S. Lowell, J. E. Shields, M. A. Thomas, M. Thommes), Springer Netherlands, Dordrecht, **2004**, pp. 15-57.

[4] aJ. Rouquerol, F. Rouquerol, in *Adsorption by Powders and Porous Solids (Second Edition)*, Academic Press, Oxford, **2014**, pp. 57-104; bP. Llewellyn, G. Maurin, J. Rouquerol, in *Adsorption by Powders and Porous Solids (Second Edition)*, Academic Press, Oxford, **2014**, pp. 565-610.

[5] K. S. Walton, R. Q. Snurr, *J. Am. Chem. Soc.* **2007**, *129*, 8552-8556.

[6] M. F. De Lange, T. J. H. Vlugt, J. Gascon, F. Kapteijn, *Micropor. Mesopor. Mater.* **2014**, *200*, 199-215.

[7] D. A. Gómez-Gualdrón, P. Z. Moghadam, J. T. Hupp, O. K. Farha, R. Q. Snurr, *J. Am. Chem. Soc.* **2016**, *138*, 215-224.
